# From Dysbiosis to Inflammation: Gut Microbiota and Oxidative Stress in Atopic Dermatitis

**DOI:** 10.3390/antiox15030299

**Published:** 2026-02-27

**Authors:** Patrycja Lipska, Kamila Łukańko, Julia Sobczak, Ivanna Lazarchuk, Anna Duda-Madej

**Affiliations:** 1 Faculty of Medicine, Wroclaw Medical University, Ludwika Pasteura 1, 50-367 Wrocław, Poland; 2 University Clinical Hospital in Wroclaw, Borowska 213, 50-556 Wrocław, Poland; 3 Provincial Specialist Hospital in Wroclaw, H. M. Kamieńskiego 73A, 51-124 Wrocław, Poland; 4 Department of Microbiology, Faculty of Medicine, Wroclaw Medical University, Chałubińskiego 4, 50-368 Wrocław, Poland

**Keywords:** atopic dermatitis, gut microbiota, dysbiosis, oxidative stress, inflammation, skin-gut axis, reactive oxygen species, antioxidant defence, immune regulation, leaky gut

## Abstract

Atopic dermatitis (AD) is a chronic inflammatory dermatosis with a complex etiopathogenesis that, despite extensive research, remains incompletely understood. The disorder affects a substantial proportion of the global population and is associated with a significant clinical burden. In recent years, increasing attention has been directed toward the gut microbiota as a potential modulator of the course of inflammatory diseases, including AD. The aim of this review is to critically examine current evidence regarding the association between gut dysbiosis and the exacerbation of inflammatory processes observed in AD. Available studies suggest that alterations in gut microbiota composition may lead to dysregulation of the gut–skin axis, increased intestinal barrier permeability, and activation of pro-inflammatory mechanisms, thereby contributing to the amplification of AD symptoms. Overall, the analyzed findings suggest that the gut microbiota represents a significant yet underexplored component of AD pathogenesis, and that its modulation may define a novel direction for future therapeutic strategies. Elucidating the mechanisms underlying the gut–skin axis may not only inform the development of preventive approaches targeting gut microbiota regulation but also support a broader view of AD as a systemic disorder in which redox imbalance is critically involved.

## 1. Introduction

The gut microbiota, a complex and dynamic community of bacteria, archaea, viruses, and fungi inhabiting the gastrointestinal tract, plays a key role in maintaining systemic homeostasis. Under physiological conditions, it contributes to: (i) digestion and metabolism of nutrients [1]; (ii) synthesis of short-chain fatty acids (SCFAs) [2]; (iii) modulation of the immune response [3]; (iv) production of neurotransmitters influencing cognitive function [4,5]; and (v) strengthening of the intestinal barrier [6,7]. Owing to these multifaceted functions, the gut microbiota affects not only local immunity but also systemic immune resilience through interactions within the gut–organ axes [8,9]. Therefore, maintaining its quantitative and qualitative balance is essential for human health.

Disruption of the compositional and functional equilibrium of the gut microbiota is referred to as dysbiosis. Important factors contributing to its development include: (i) endogenous factors, such as an unhealthy diet deficient in dietary fiber, polyphenols, and SCFAs [10]; (ii) excessive or inappropriate antibiotic therapy, which induces long-lasting alterations in microbiota composition and diversity [11,12]; (iii) psychosocial factors, including chronic stress [13,14]; (iv) environmental factors, such as air pollution, heavy metals, pesticides, and food additives [15,16,17]. The accumulation of these factors can induce chronic oxidative stress within the gut and, through disruption of redox balance, lead to damage to the intestinal epithelium and activation of inflammatory signaling pathways [18,19]. These processes are directly associated with a weakened intestinal mucosal barrier, increased intestinal permeability (“leaky gut”), and translocation of bacterial cells and their metabolites (e.g., toxins and/or lipopolysaccharide) into the systemic circulation [20,21]. Consequently, both primary and secondary immune responses of the host are stimulated [22], activating inflammatory pathways including NF-kB, MAPK, and NLR3, leading to the production of pro-inflammatory cytokines and chemokines [23,24,25]. This dysregulation of the gut–skin axis may lead to the initiation and progression of atopic dermatitis (AD), as evidenced by a growing number of studies reported in the literature.

Patients with AD often present with reduced gut microbiota diversity, decreased beneficial genera such as *Bifidobacterium* and *Lactobacillus*, and an increased presence of potentially pathogenic species, i.e., *Escherichia* and *Clostridium* [26,27,28]. These alterations correlate directly with disease severity and elevated levels of inflammatory markers. Moreover, studies have shown that dysbiosis can disrupt the Th1/Th2 balance and promote polarization towards the Th2 profile characteristic of atopic conditions [29,30,31]. Undoubtedly, oxidative stress further exacerbates this pathological cascade through imbalance between the production of reactive oxygen species (ROS) and the host’s antioxidant defense mechanisms [32,33]. This situation, in turn, promotes lipid peroxidation, protein and DNA damage, and the activation of transcription factors such as NF-kB, thereby amplifying pro-inflammatory cytokine production [34,35]. Clinical studies involving patients with AD have demonstrated elevated levels of oxidative stress markers with simultaneous weakening of antioxidant capacity, which directly correlated with the intensity of skin lesions [33,36,37,38]. Collectively, these findings provide compelling evidence for the involvement of oxidative stress and gut microbiota dysbiosis in the dysregulation of the gut–skin axis, in which the intestinal microbiota plays a key role. Its members contribute to metabolic changes, thereby affecting skin barrier integrity, dendritic cell activation, Tcell polarization, and the production of inflammatory mediators. This complex, multi-level mechanism translates into the clinical severity of AD symptoms, its frequent relapses, and its correlation with other atopic diseases, such as asthma and allergic rhinitis [39,40,41].

However, despite extensive research leading to an understanding of the pathogenesis of AD, a substantial gap remains in the comprehensive clinical integration of gut dysbiosis, oxidative stress, and inflammatory mediators within a unified mechanistic framework. This review aims to synthesize the current state of knowledge regarding the impact of gut microbiota disturbances on the development and progression of AD. Its novelty lies in its particular focus on the role of oxidative stress as a critical mechanistic link connecting dysbiosis with chronic inflammation in AD.

## 2. Pathophysiology of Atopic Dermatitis

AD is a complex disease entity and one of the most frequently encountered chronic inflammatory skin disorders. Despite its high prevalence, the pathophysiology of AD remains incompletely understood. Among the predisposing factors, genetic and environmental influences, dysfunctions of the skin barrier, and dysregulation of the immune response are considered to play key roles [42,43,44]. Although the subsections of this part of the manuscript are distinguished to facilitate clarity and organization, it is important to emphasize that the factors discussed are highly interdependent, and their effects overlap and interact, ultimately contributing to the development of AD.

### 2.1. Genetic Determinants

Among the genetic abnormalities predisposing individuals to the development of AD, mutations that disrupt filaggrin function are highlighted most prominently [45,46,47,48]. Filaggrin (FLG) is a structural, histidine-enriched protein that, in healthy skin, maintains cellular integrity [49]. The precursor of FLG is profilaggrin, which forms keratohyalin granules (KHGs) within the cytoplasm of the granular layer (predominantly) and the spinous layer. These granules are integral to epidermal keratinization through their interaction with keratin filaments and promotion of their cross-linking. This process ensures the cohesion of the epidermal barrier and enables the separation of the internal milieu of the human body from the external environment [50]. Mutations in FLG are widely recognized as a major contributor to both the onset and chronicity of AD [49,51]. These FLG mutations are often accompanied by impairments in epidermal barrier integrity, which facilitate exposure to allergenic factors and predispose the skin to bacterial colonization [52,53]. Although most patients with AD exhibit impairment of the skin barrier, fewer than one-third carry FLG null mutations that result in loss of function. Well-characterized mutations include R501X and 2282del4 [54]. Importantly, reduced levels of FLG are also observed in patients with AD who do not carry FLG mutations, indicating that this protein represents a fundamental factor underlying the pathogenesis of the disease [48,55]. It is worth emphasizing that FLG in the stratum corneum (SC) undergoes degradation into free amino acids—pyrrolidone carboxylic acid (PCA) and urocanic acid (UCA)—which are pivotal for maintaining proper skin hydration, photoprotection, and the regulation of immune responses. These acids represent fundamental elements of natural moisturizing factor (NMF) [56,57]. A study assessing the relationship between AD severity, FLG genotype, and levels of FLG degradation products demonstrated that reduced NMF constitutes a principal component of moderate-to-severe disease [57]. Furthermore, carriers of FLG null mutations exhibit significantly decreased NMF levels throughout the full thickness of the SC, along with increased transepidermal water loss (TEWL) and skin dryness, a hallmark feature of AD [58,59].

### 2.2. Skin Barrier Dysfunction and Skin Dysbiosis

Human skin is often described as the largest organ of the body, harboring a highly abundant and heterogeneous microbial community [60,61,62]. The vast majority of colonizing microbes are harmless to the host. Moreover, symbiotic colonization can provide numerous benefits. The skin microbiota may participate in regulating its metabolism and function as a primary defensive barrier, protecting against the expansion of detrimental microorganisms [62,63,64]. Microorganisms classically inhabiting the skin include fungi of the genus *Malassezia* and bacteria such as coagulase-negative staphylococci (namely *Staphylococcus epidermidis*, *Staphylococcus lugdunensis*, and *Staphylococcus hominis*), as well as members of the families *Streptococcaceae*, *Cutibacteriaceae*, and *Corynebacteriaceae* [65]. However, under certain conditions, particularly in the case of AD, skin dysbiosis can occur. By definition, skin dysbiosis is a disruption of the balance of the microbiome residing in this organ [63,66]. This leads to the absence or deficiency of regularly occurring microorganisms, accompanied by the presence of pathogenic microbes, which collectively produce adverse effects for the host [67]. A reduction in the richness of the microbiome in lesional skin compared to healthy skin is characteristic of AD [68]. Numerous studies have shown that enhanced colonization by *Staphylococcus aureus* in AD leads to a reduction in commensal skin bacteria and results in diminished synthesis of antimicrobial peptides (AMPs), which are normally produced by the skin to inhibit the growth of pathogenic microorganisms [69,70,71]. Disruption of AMP production may therefore elevate the risk of viral, bacterial, and fungal skin infections, consequently leading to a deterioration in patients’ quality of life [71]. Table 1 summarizes a comparison of the changes occurring in the populations of microorganisms inhabiting the skin of individuals diagnosed with AD.

The skin barrier serves as a bridge in the complex interplay among the skin microbiota, environmental factors, genetic determinants, and the host immune system [49]. The integrity of this barrier is maintained by the epidermis, which undergoes a process known as keratinization or cornification. Histologically, the epidermis consists of four or five layers, varying by location in the body [50]. The outermost layer is the stratum corneum (SC). Moving inward, the epidermis comprises the stratum lucidum—typically found only on the soles of the feet and the palms—followed by the granular layer, the spinous layer, and the basal layer [50,52]. Barrier dysfunction affects the majority of patients with AD and is a characteristic feature of the disease [54,73]. The barrier’s function is primarily determined by the SC, where the stage of keratinization occurs—keratinocytes undergo enucleation and flattening to become corneocytes, and their cell membranes are substituted by the cornified envelope [74,75]. A disrupted epidermal barrier increases susceptibility to irritation and facilitates the penetration of exogenous antigens into the skin, where they interact with resident immune cells and trigger inflammation [59,74,76]. Conversely, prolonged skin inflammation further impairs the epidermal barrier, highlighting the significance of preserving barrier integrity for both the treatment and prevention of allergic diseases [74].

### 2.3. Environmental Determinants

Given that the skin serves as a defense against agents such as toxins and UV light, it is intriguing to consider how external environmental factors, including individual behaviors, influence the course of AD [77]. Among environmental factors, chemicals such as preservatives, harsh detergents, and fragrances are recognized as contributing to the pathophysiology of AD. The use of strong, alkaline cleansing agents can disrupt skin pH and promote local inflammation [42]. Even water may decrease FLG levels in the epidermis, thereby disrupting the structure of a functional skin barrier [55]. A compromised skin barrier is particularly susceptible to environmental factors, which in AD exacerbate inflammation, leading to secondary damage to the SC and perpetuating the disease’s vicious cycle [73]. In addition to the factors mentioned above, environmental pollution also seems to influence the occurrence of AD [76]. Exposure to air pollution in the place of residence was associated with an elevated risk of developing AD exclusively in males, as well as a higher likelihood of allergic response to airborne allergens in both males and females [78]. Moreover, gases such as sulfur dioxide, nitrogen dioxide, and carbon monoxide, as well as particulate matter present in the air, have been classified as independent contributors to AD [79]. Furthermore, exposure to particulate matter experimentally suppressed FLG expression in keratinocytes in vitro via TNF-α(tumor necrosis factor-alpha), resulting in increased TEWL [80]. The collected data clearly indicate that broadly defined environmental factors—from chemical to atmospheric—can significantly disrupt skin barrier integrity and thereby contribute to the development and exacerbation of AD.

### 2.4. Lifestyle Factors in the Pathophysiology of AD

Tobacco smoking is considered a factor that may increase the prevalence of AD [81]. A genetic correlation has even been observed between tobacco use and multiple allergic conditions, particularly asthma [82]. To investigate the association of exposure to smoking and the occurrence of allergic diseases in children, such as asthma and AD, a study was conducted that included control data from a total of 53,505 children [83]. Notably, both maternal smoking during pregnancy and passive smoke exposure were linked to childhood asthma, whereas no statistically significant relationship was found between contact with tobacco and the development of AD in children [83]. A more recent meta-analysis from 2024, however, reveals that AD in offspring is not connected with active maternal smoking during pregnancy, but is nevertheless correlated with passive tobacco exposure [84]. Another study assessing the relationship between active and passive smoking and the occurrence of AD focused on an older population—Korean adolescents. A positive connection was observed regarding AD prevalence and both the frequency and intensity of contact with each type of smoking [85]. Exposure to tobacco smoke during childhood may result in AD onset in adulthood [86]. Interestingly, a secondary cross-sectional analysis from 2018 demonstrated that both the type of smoking and the sex of the participant influence the prevalence of AD in adults. It was found that, in women, AD incidence was associated with current tobacco use, whereas in men, no correlation was observed between either active or passive smoking and AD occurrence [87].

Sleep disturbances are a common comorbidity among individuals with AD, affecting approximately 47% to 80% of the pediatric population and 33% to 90% of the adult population [88,89]. Interestingly, impaired sleep quality appears to be particularly pronounced among adult patients, males, and individuals with severe forms of AD, which is generally associated with greater difficulty maintaining restorative sleep throughout the night than with sleep initiation [90]. Among pediatric patients, pruritus is the predominant symptom reported as a cause of sleep disruption. The consequences of sleep disturbances in children with AD may include behavioral disorders, short stature, cognitive dysfunction, and mood fluctuations [91]. The inflammatory state present in AD may also contribute to the development of additional psychiatric disorders, as pro-inflammatory cytokines are elevated not only locally in the skin but also throughout the body. Moreover, sleep is widely recognized as a central regulator of immune system function. In addition, sleep disturbances are frequently accompanied by increased psychological stress [92]. Stress is a well-established factor that exacerbates the course of various diseases, including AD [93]. Stress may lower the itch threshold, leading to nighttime scratching, which further worsens the already-compromised skin barrier [92]. Stress itself may also impair skin barrier function by stimulating the secretion of pro-inflammatory cytokines, delaying wound healing, and aggravating dermatological conditions such as AD, acne, psoriasis, and urticaria [93]. Although there is growing evidence suggesting that psychological stress plays a significant role in the onset and exacerbation of AD, the underlying pathophysiological mechanisms have not been fully elucidated [94].

### 2.5. Deregulation of Immune Responses

**Figure 1:**
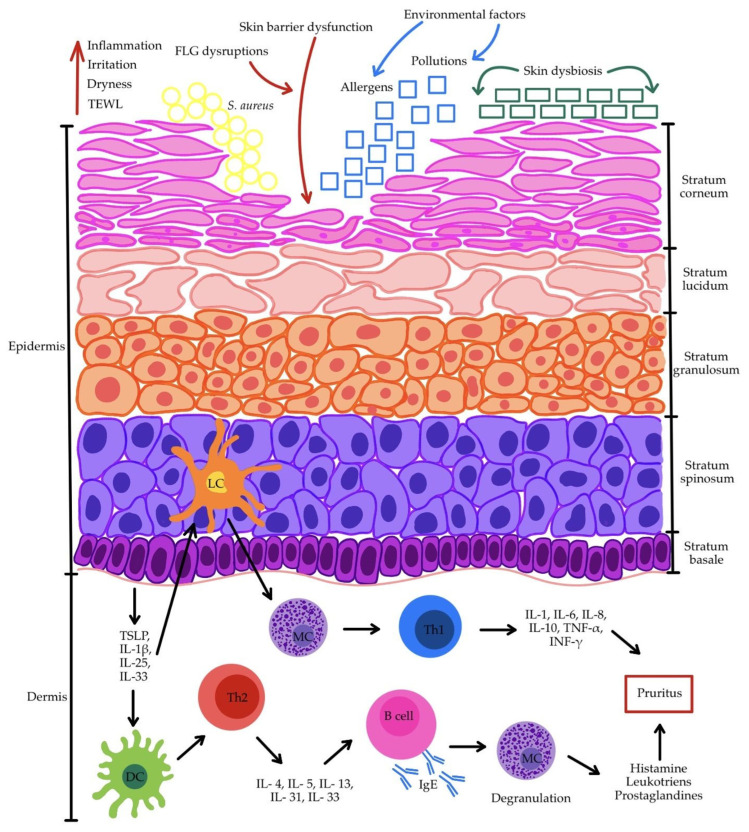
Multifactorial pathogenesis of AD. Abbreviations: B cell—B lymphocyte; DC—dendritic cell; FLG—filaggrin; IFN-γ—interferon gamma; IgE—immunoglobulin E; IL-1—interleukin 1; IL-1β—interleukin 1 beta; IL-10—interleukin 10; IL-13—interleukin 13; IL-25—interleukin 25; IL-31—interleukin 31; IL-33—interleukin 33; IL-4—interleukin 4; IL-5—interleukin 5; IL-6—interleukin 6; IL-8—interleukin 8; LC—Langerhans cell; MC—mast cell; *S. aureus*—*Staphylococcus aureus*; TEWL—transepidermal water loss; Th1—T helper 1 lymphocyte; Th2—T helper 2 lymphocyte; TNF-α—tumor necrosis factor-alpha; TSLP—thymic stromal lymphopoietin. Symbols: yellow circles indicate *S. aureus* colonization; blue arrows and blue squares represent environmental factors such as allergens and pollutants penetrating the skin; green rectangles denote disrupted skin microbiota composition (skin dysbiosis); red arrows show the effect of impaired skin barrier on the skin.

Immune system dysfunctions constitute a significant component of AD pathophysiology. The primary complex abnormality consists of dysregulation of Th1/Th2, Th17, and Treg cell responses. In the acute phase of AD, the immune response is predominantly Th2-driven, whereas in the chronic phase, Th1, Th17, and Th22 responses prevail [53,95,96]. Langerhans cells (LCs) in the epidermis function as the key immune cells, whereas deeper within the dermis, macrophages, T cells, and dendritic cells (DCs) are present. Keratinocytes in the skin have the capacity to secrete inflammation-promoting cytokines, including TNF and the interleukins IL-1β, IL-6, IL-10, and IL-18 [69]. Keratinocytes can also synthesize thymic stromal lymphopoietin (TSLP), a pro-inflammatory cytokine that modulates Th lymphocyte activity [53]. TSLP is a principal sensitizing molecule, as it induces DCs and stimulates Th2-type immune responses, thereby amplifying inflammation and enhancing allergic reactivity [95,97]. A dysfunctional skin barrier promotes the secretion of TSLP, IL-1β, IL-25, and IL-33 by keratinocytes [98]. This stimulates LCs and DCs, triggering the activation of Th2, Th17, and Th22 immune responses. The Th1 response is induced through mast cells (MCs) that have been previously activated by LCs. Th1 lymphocytes subsequently secrete IL-1, IL-6, IL-8, IL-10, TNF-α, and IFN-γ. In contrast, Th2 lymphocytes release IL-4, IL-5, IL-13, IL-31, and IL-33 [98,99]. It is worth emphasizing that Th2-driven inflammation can affect keratinocytes maturing in the presence of cytokines and decrease FLG expression, thereby exacerbating pre-existing skin barrier dysfunction [54,73]. Moreover, cytokines produced by Th2 cells stimulate B lymphocytes to produce IgE antibodies, which in turn induce MC degranulation and the release of mediators including histamine, leukotrienes, and prostaglandins. This drives the pruritus–scratch cycle, further damaging the skin barrier [43,45,53,99]. Figure 1 provides a schematic representation of key pathophysiological mechanisms in AD.

### 2.6. Signaling Pathways and Molecular Mechanisms Involved in AD Pathogenesis

The etiology of AD is driven by complex molecular mechanisms, including activation of the Janus kinase–signal transducer and activator of transcription (JAK–STAT) signaling pathway, which constitutes a principal intracellular signaling cascade for cytokines. Four members of the JAK family have been identified—JAK1, JAK2, JAK3, and tyrosine kinase 2 (TYK2)—alongside seven members of the STAT family: STAT1, STAT2, STAT3, STAT4, STAT5A, STAT5B, and STAT6 [100]. Importantly, the JAK–STAT pathway is crucial in Th2-mediated immune responses, particularly those driven by IL-4, IL-13, and IL-31, often referred to as cytokines involved in the acute phase of AD [101]. IL-4 impairs skin barrier integrity by reducing the expression of critical proteins such as filaggrin, promoting IgE class switching in B cells, and recruiting immune effector cells, including eosinophils and mast cells [102,103]. IL-4 signals via two receptor types: type I receptors, formed by the IL-4Rαchain and the common γ-chain, and type II receptors, comprising IL-4Rα and IL-13 receptor α1 (IL-13Rα1) chains [100]. Type II IL-4 receptor complexes are predominantly expressed on non-hematopoietic cells, such as keratinocytes, fibroblasts, and immune cells, where they contribute significantly to AD development. In contrast, type I IL-4 receptor complexes are primarily restricted to hematopoietic cells. Additionally, type II IL-4 receptors function as IL-13 receptors, with IL-13 representing a key mediator of pruritus that links immune and neural pathways [102]. In the type I IL-4 receptor-mediated pathway, JAK1 and JAK3 are activated by phosphorylation, subsequently leading to STAT6 signaling. Conversely, stimulation of the type II IL-4 receptor complex results in JAK1 and TYK2 activation, with JAK1 linked to the IL-4Rα chain and TYK2 associated with the IL-13Rα1 chain. These events are followed by STAT6 and STAT3 induction, which ultimately upregulate the expression of genes driving type 2 inflammation [100,102]. IL-31 receptors can also exist in two forms, including a heterodimer composed of the IL-31 receptor αchain (IL-31RA) and the oncostatin M receptor βchain (OSMRβ) [100]. Through the activation of DCs, IL-4, IL-13, and IL-31 promote the production of chemokines, such as CCL17, CCL22, and CCL26, thereby recruiting CCR4+ Th2 cells to sites of inflammation [102]. TSLP also represents a crucial cytokine in the initiation of type 2 immune responses. In AD, it is highly expressed in cutaneous epithelial cells and, together with IL-33, is further released from keratinocytes upon skin barrier disruption, induced by scratching [101]. In turn, TSLP activates transient receptor potential (TRP) sensory neurons, contributing to further pruritus. Additionally, it stimulates keratinocytes to release periostin and IL-33, which in turn enhance IL-13 and IL-31 production, thereby sustaining the itch–scratch cycle [102,103]. Moreover, TSLP promotes OX40L expression on DCs, a critical signal for the polarization of naïve T cells toward a Th2 phenotype [102].

## 3. Gut Dysbiosis in Atopic Dermatitis

### 3.1. The Core Composition of the Human Gut Microbiome

The human gut is colonized by more than 100 trillion microorganisms, including bacteria, archaea, fungi, and viruses, forming a complex and diverse ecosystem [28,104]. Altogether, gut bacteria weigh approximately 1–1.5 kg [28]. The predominance of bacteria residing in the adult gut is attributed to two bacterial phyla: the Gram-negative *Bacteroidetes* and the Gram-positive *Firmicutes* [28,105]. More than 99% of bacteria comprise the *Firmicutes*, *Bacteroidetes*, *Proteobacteria*, and *Actinobacteria* [28]. Other bacterial phyla, present at subdominant levels in the human gut, vary significantly among individuals. These include the *Actinobacteria*, *Fusobacteria*, and *Verrucomicrobia* phyla [105]. *Verrucomicrobia*, for instance, is more frequent in children aged 3–12 years [106]. *Bifidobacterium* is among the first bacteria to colonize the human gut and is a dominant member of the intestinal microbiota during breastfeeding, typically for at least the first 3 to 4 months [107].

### 3.2. The Role of SCFAs and Bacterial Dysbiosisin Atopic Dermatitis Development

**Figure 2:**
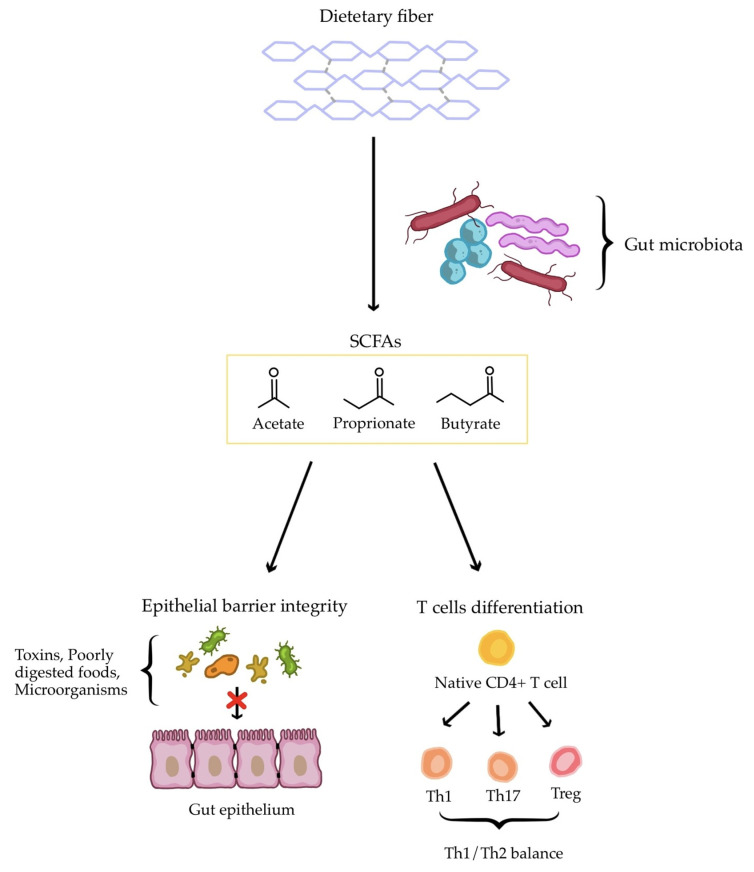
The role of SCFAs in the human gut and immune system (simplified scheme). Abbreviations: SCFAs—short-chain fatty acids; Th1—T1 helper lymphocyte; Th17—T17 helper lymphocyte; Treg—regulatory T lymphocyte.

From the first month of life, the gut microbial community is essential for the maturation of the human immune system. This is closely linked to the production of various metabolites and signal molecules, such as post-translationally modified peptides, amino acid metabolites, short-chain fatty acids, oligosaccharides, glycolipids, and non-ribosomal peptides, all of which influence the systemic immune response [105]. A healthy gut microbiota supports the Th1/Th2 balance by stimulating the development of Th1 cells. Overall, the main factors influencing gut health include the level of microbial diversity, an abundance of SCFA producers, and the presence of potentially pathogenic representatives [106]. A reduction in bacterial species diversity may be associated with dysbiosis, further contributing to elevated inflammation. The onset and severity of AD are thought to correlate with the degree of gut dysbiosis. Moreover, patients with AD have been reported to have reduced levels of SCFAs [105,108]. These acids, including butyrate, propionate, acetate, and lactate, originate from fermentation of fiber by gut microbiota. They play an important role in preserving gut epithelial barrier integrity. Lower SCFA levels promote easier penetration of toxins, undigested food particles and gut microorganisms into the systemic circulation, thereby aggravating skin inflammation [105]. SCFAs also enhance innate immunity, improving the skin’s defense against inflammation and supporting adaptive immunity. This occurs through influencing T and B cell differentiation [109], particularly by regulating the production of Th1, Th17, and regulatory T cells (Tregs) [106,110]. Among SCFAs, butyrate is crucial for mucosal Treg differentiation [110]. Dysregulation of Treg cells or an imbalance in cytokines, especially elevated pro-inflammatory cytokines such as IL-6 and IL-17, can contribute to the onset and progression of inflammatory dermatoses [106,110]. The interplay between SCFAs, the intestinal barrier, and systemic immune modulation is summarized in Figure 2 (simplified scheme).

### 3.3. The Infant Gut Microbiome and SCFA Producers

The highest incidence of AD occurs during infancy (the first 12 months of life), extending to early childhood [111]. The positive correlation between the poor diversity of gut microbiota among infants and the prediction of AD onset has been demonstrated in various studies [108,112,113]. It has been shown that infants with AD have significantly less-diverse gut microbiota and higher levels of *Clostridoides difficile*, *Escherichia coli* and *Bacteroides* spp., compared to healthy controls [114,115]. Microbes from the *Clostridium* genus might aggravate the level of inflammation via eosinophilic inflammation [115]. Increased abundances of *Clostridiaceae* are further associated with excessive toxin release, which inhibits the chemotaxis of neutrophils and suppresses eosinophil activity, thereby promoting gut inflammation, as shown in a study examining AD risk across 24 cohorts [116]. In a different study [117], species such as *C. difficile* and pathogenic *E. coli* were linked to increased intestinal epithelial barrier leak in infants. “Leaky gut syndrome” is also implicated in insufficient SCFA production [105]. Inadequate production might be due to suppression of SCFA-producing bacteria by pathogenic species. Notably, some *Clostridiales*, like *Clostridiales* Family XIII Incertae Sedis, which produce SCFAs, show reduced numbers in AD patients [106]. In a study [108] by Alam et al., preschool-aged children who were at the highest risk of developing atopy exhibited lower initial levels of particular bacterial genera, including *Bifidobacterium*, *Akkermansia*, and *Faecalibacterium*, measured during infancy. This largely coincides with the results of research by Moniaga et al., where reduced levels of *Akkermansia*, *Lactobacillus*, *Faecalibacterium prausnitzii*, and *Bifidobacterium* were specific to AD patients [105]. Importantly, *Akkermansia*, *Bifidobacterium*, and *Faecalibacterium* are producers of SCFAs [116]. A decreased abundance of *Eubacteriaceae* among atopic patients is strongly associated with impaired butyrate production. Additionally, a higher abundance of *Eubacteriaceae* has been linked to lower levels of the pro-inflammatory mediators eotaxin/CCL11 (eosinophil chemotactic protein/C-C motif chemokine 11), MDC/CCL22 (macrophage-derived chemokine/C-C motif chemokine 22), and Flt3L (fms-related tyrosine kinase 3 ligand), which are involved in the Th2 response [106].

### 3.4. The Varying Relevance of Different Bacterial Strains Belonging to the Same Genus

Reduced levels of the *Bifidobacteria* genus might be influenced by a lack of breastfeeding [118,119,120]. The *Bifidobacterium* genus is generally considered a protective factor against inflammatory dermatoses through the production of SCFAs in murine models [110]. Additionally, γ-aminobutyric acid produced by *Lactobacillus* and *Bifidobacterium* in the gut has been shown to suppress skin itch [105]. However, a higher incidence of certain *Bifidobacteria* species, including *Bifidobacterium catenulatum* and *Bifidobacterium pseudocatenulatum*, has been reported to impact AD development across different age groups [108,121]. In another study, Depner et al. used 16S rRNA amplicon sequencing of fecal samples [107]. It was found that during the first two months of life, members of *Bifidobacterium* were dominant and showed the strongest and most consistent inverse associations with AD. Species such as *Bifidobacterium longum* and *Bifidobacterium bifidum*, which possess the specific ability to digest human milk oligosaccharides (HMOs) in breastmilk and to produce beneficial aromatic lactic acids, were most prevalent at two months of age. Among AD patients, aged 2–12 months, the composition of the *Bifidobacterium* genus changed profoundly, showing a premature shift toward adult-like *Bifidobacterium* spp., especially *Bifidobacterium adolescentis* and *B. catenulatum*. This phenomenon suggests that the protective effect of *Bifidobacterium* against AD depends on a critical window and an adequate combination of species [107].

Although previously cited studies highlight a reduction in the abundance of *F. prausnitzii* among AD patients [105,108], evidence also indicates that their numbers may be elevated in this group [120,122]. *F. prausnitzii* is the main producer of SCFAs in healthy individuals. However, subspecies of *F. prausnitzii*, insufficient in SCFA production, are more common among AD patients [123]. This could explain potential differences in research results. In addition, genes encoding carbohydrate-active enzymes (CAZymes), which break down SCFA-resistant starch, are insufficiently represented in the intestinal microbiota of patients with AD [124].

### 3.5. The Potential Relationships Between Immune Response Modulators and Gut Microbiota

Other bacteria, including *Veillonellaceae*, *Fusicatenibacter*, *Flavonifractor*, and *Odoribacter*, have been identified as potentially protective factors against inflammatory dermatoses, possibly through effects on circulating inflammatory cytokines, as shown in a Mendelian randomization study. Cytokine IL-15RA was identified as an important mediator linking the bacterial family *Veillonellaceae* to eczema. It triggers the MAPK signaling pathway and promotes the secretion of pro-inflammatory cytokines, such as IL-6, IL-8, and TNF-α. *Fusicatenibacter* and *Odoribacter* help maintain immune balance by regulating IL-10 production and Treg differentiation. Elevated abundance of *Flavonifractor* has been observed in moderate-to-severe AD, indicating its potential role in immune dysregulation [109]. A different study conducted by Kalashnikova et al. analyzed the potential links between the microbial community diversity and cytokine levels in the blood serum of children [106]. Among the atopic group, a significant increase in the levels of several markerscharacteristic of the Th2 response was observed. These include MDC/CCL22, IL-5, IL-8, IL-13, IFN-γ (interferon gamma), TNF-α, MIP-1α/CCL3 (macrophage inflammatory protein 1-α/chemokine (C-C motif) ligand 3), and VEGF (vascular endothelial growth factor). On the contrary, atopic patients exhibited reduced levels of IL-2, IL-1α, IL-15, and IL-17A [106]. There is also a causal relationship between IL-18R1 and an increased risk of eczema. IL-18R is expressed on various immune cells, among which are Th1, NK, and mast cells, suggesting a broad immunomodulatory function [109]. In another study [123], it was observed that children with more severe AD, assessed using the SCORAD (SCORing Atopic Dermatitis) index, had higher fecal calprotectin levels, as well as elevated blood eosinophils and IgE levels, indicating increased inflammatory status of the gastrointestinal tract. Additionally, the concentrations of IL-10 and IFN-γ in the bloodstream, influenced by intestinal microbes, alter levels of cortisol, a stress hormone. In turn, higher cortisol levels can negatively affect gut microbiota composition and intestinal barrier permeability, creating a vicious cycle [105]. MDC/CCL22 is a key mediator of atopic disorders. A positive association was found between the *Pasteurellaceae* family and MDC/CCL22 levels. On the other hand, a negative correlation was observed between MDC/CCL22 levels and bacterial taxa, such as *Barnesiellaceae*, *Oscillospiraceae*, *Peptococcaceae*, *Eubacterium coprostanoligenes*, and *Clostridia* UCG-014, indicating possible protective effects of these species. Notably, in the same study, atopic patients showed a significant increase in the abundance of *Pasteurellaceae*, *Peptococcaceae*, and *Clostridia* UCG-014, whereas decreases were observed in other groups, including *Barnesiellaceae*, *Eubacteriaceae*, *Clostridiales* Family XIII Incertae Sedis, *Oscillospiraceae*, *Anaerovoraceae*, and *Flavobacteriaceae*, compared to the control group [106]. According to an analysis of a dataset from 24 cohorts, *Lachnospiraceae* UCG001 strains exacerbate gut dysbiosis, which was further correlated with impaired amino acid metabolism, increased blood uric acid levels, and CD4 TH17-driven inflammation [116]. The varying effects of specific bacterial strains are summarized in Table 2 and Table 3, which present microorganisms with potential protective or adverse impacts on atopic dermatitis development.

The results of the studies reviewed indicate that the relationship between the microbiota and the immune system is highly complex, providing multiple potential targets for both the diagnosis and treatment of eczema.

## 4. Gut Microbiota and Oxidative Stress in Atopic Dermatitis

### 4.1. Gut Microbiota, Dysbiosis and Immune Homeostasis

The gut microbiota constitutes a highly dynamic and metabolically active ecosystem that plays a central role in shaping immune tolerance, regulating oxidative balance, and maintaining epithelial barrier function [125]. Perturbations in its composition, generally referred to as dysbiosis, are increasingly recognized as a driving factor in the initiation and persistence of chronic inflammatory disorders, including AD [126]. Mounting evidence demonstrates that dysbiosis not only disrupts host immune equilibrium but also profoundly influences redox homeostasis, thereby fueling a self-sustaining loop of oxidative stress and inflammation that contributes to the chronicity and exacerbation of cutaneous pathology in AD. One of the most critical consequences of gut dysbiosis is the loss of beneficial commensals with anti-inflammatory and antioxidant properties, notably *F. prausnitzii* and various *Bifidobacterium* species, accompanied by the expansion of pathobionts such as *Enterobacteriaceae* and *Clostridium perfringens* [96,122,127]. This compositional shift leads to profound disturbances in microbial metabolism.

### 4.2. Microbial Metabolites and Redox Regulation

Under healthy conditions, commensals ferment dietary fibers into SCFAs, particularly butyrate, propionate, and acetate. Butyrate is especially relevant because of its ability to serve as an energy source for colonocytes, reinforce intestinal barrier integrity, and exert potent immunoregulatory and antioxidant effects [128]. Mechanistically, butyrate activates the Nrf2 signaling pathway, which upregulates the transcription of antioxidant response elements, including superoxide dismutase, catalase, and glutathione peroxidase [129,130]. In addition, SCFAs modulate epigenetic regulation through histone deacetylase inhibition, promoting the differentiation and stability of Tregs. A deficiency in SCFAs therefore results in impaired Treg function, a skewing toward pro-inflammatory Th2 and Th17 responses, and the excessive release of cytokines such as IL-4, IL-5, IL-13, IL-17, and IL-22, which directly stimulate ROS generation and tissue damage [131,132]. Tryptophan metabolism constitutes another critical link between dysbiosis and oxidative stress [133]. Commensal bacteria are key contributors to the conversion of tryptophan into immunomodulatory metabolites, including indole derivatives and kynurenine pathway intermediates, which activate the aryl hydrocarbon receptor (AhR). AhR signaling is central to the maintenance of epithelial barrier integrity and redox homeostasis. In dysbiotic states, the reduced microbial capacity for tryptophan catabolism results in lower levels of these protective metabolites, leading to increased susceptibility to oxidative stress and loss of mucosal tolerance.

### 4.3. Bidirectional Interaction and Clinical Evidence

Oxidative stress itself reinforces dysbiosis, thus establishing a bidirectional pathogenic relationship. Elevated levels of ROS damage microbial DNA and proteins, generating mutagenic byproducts and selecting for bacteria with enhanced stress-resistance phenotypes [134]. ROS also alter luminal redox potential, suppressing obligate anaerobes such as butyrate producers and creating a niche favorable for facultative anaerobes and opportunistic pathogens, which thrive under oxidative pressure [135]. At the epithelial level, ROS impair mitochondrial function and increase lipid peroxidation, weakening epithelial tight junctions and mucosal defenses. The loss of barrier integrity permits further microbial translocation and antigen exposure, which sustains immune activation and accelerates ROS production, thereby perpetuating the cycle of inflammation and dysbiosis [136]. Clinical and experimental evidence support this bidirectional relationship. Biomarkers of oxidative stress, such as 8-hydroxy-2′-deoxyguanosine (8-OHdG), advanced oxidation protein products, and malondialdehyde (MDA), show strong correlations with disease severity in AD [36]. Parallel microbiota profiling in AD cohorts consistently demonstrates a reduction in SCFA-producing taxa and an overrepresentation of inflammation-associated bacteria. In murine models, antibiotic-induced dysbiosis has been shown to exacerbate AD-like skin inflammation by depleting SCFAs, confirming the causal role of microbial imbalance in linking gut metabolism to cutaneous inflammation [36].

### 4.4. Gut–Skin Axis and Oxidative Stress

**Figure 3:**
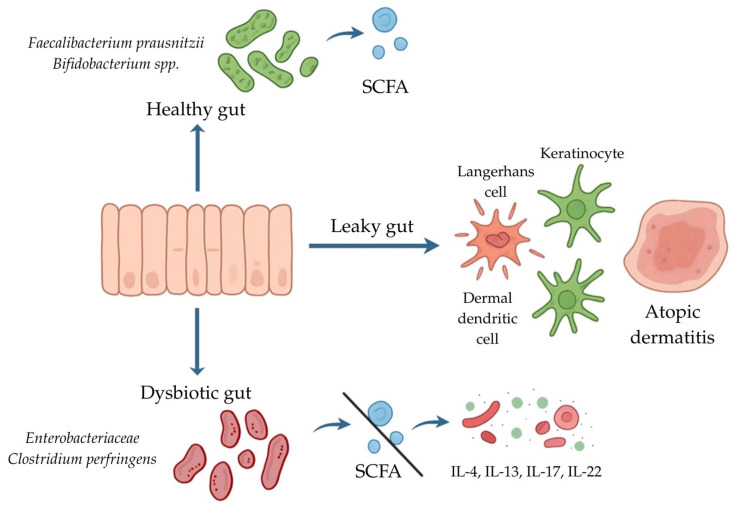
Proposed gut–skin axis in AD. In a healthy gut, commensal bacteria such as *F. prausnitzii* and *Bifidobacterium* spp. produce SCFAs that support intestinal barrier integrity and immune homeostasis. Dysbiosis, characterized by the overgrowth of *Enterobacteriaceae* and *C. perfringens*, reduces SCFA production and contributes to increased intestinal permeability (“leaky gut”). This facilitates immune dysregulation, leading to activation of dendritic and Langerhans cells in the skin, elevated levels of pro-inflammatory cytokines (IL-4, IL-13, IL-17, IL-22), and the development of AD. Abbreviations: IL-13—interleukin 13; IL-17—interleukin 17; IL-22—interleukin 22; IL-4—interleukin 4; SCFA—short-chain fatty acid.

A crucial pathophysiological mechanism by which gut-derived oxidative and inflammatory signals influence cutaneous tissues is the phenomenon of increased intestinal permeability, commonly described as “leaky gut” [36]. ROS-induced epithelial injury, compounded by pro-inflammatory cytokine activity, disrupts tight junction proteins such as claudins, occludin, and zonula occludens-1. As a result, microbial components including lipopolysaccharide (LPS), peptidoglycan, flagellin, and other metabolites translocate into systemic circulation [99]. These pathogen-associated molecular patterns (PAMPs) activate toll-like receptors (TLRs) and nucleotide-binding oligomerization domain (NOD)-like receptors on innate immune cells, amplifying systemic inflammation. Circulating inflammatory mediators subsequently act on cutaneous immune cells, including keratinocytes, Langerhans cells, and dermal dendritic cells, thereby extending the inflammatory process from the gut to the skin [96]. This gut–skin crosstalk establishes a mechanistic basis for the systemic propagation of inflammation in AD. From a therapeutic perspective, these insights underscore the potential of interventions aimed at restoring eubiosis and reducing oxidative stress. Probiotic and prebiotic supplementation has been shown to increase SCFA levels, strengthen epithelial barrier function, and suppress ROS accumulation [137]. Strains such as *Lactobacillus rhamnosus* GG and *B. longum* have demonstrated particular efficacy in modulating immune responses and reducing AD severity. Additionally, dietary interventions rich in fermentable fibers and polyphenols support SCFA production and enhance antioxidant defenses. Parallel strategies targeting oxidative stress, such as the use of N-acetylcysteine, vitamin E, or polyphenolic compounds (e.g., resveratrol, quercetin), have shown efficacy in reducing oxidative biomarkers and ameliorating clinical symptoms [36]. Importantly, combinatory approaches integrating microbiota modulation with antioxidant supplementation may prove superior in breaking the vicious cycle of dysbiosis and oxidative stress. The proposed mechanisms linking intestinal dysbiosis, oxidative stress, and skin inflammation in atopic dermatitis are summarized in Figure 3.

### 4.5. Impact of Skin Microbiota on Gastrointestinal Homeostasis

The gut–skin axis is a complex bidirectional communication network in which disruption of the skin barrier and cutaneous responses to environmental stimuli significantly impact systemic immunity and intestinal health [125]. While traditional research has focused on the gut’s influence on the skin, recent experimental mouse models demonstrate that dermal injury or the digestion of dermal hyaluronan directly alters the gut microbiome and disrupts intestinal immune homeostasis [138]. Specifically, skin wounding in mice or hyaluronidase activation leads to the systemic circulation of hyaluronan (HA) fragments, which act as damage-associated molecular patterns. These skin-derived signals trigger the increased expression of host defense genes in the colon, such as Reg3 and Muc2; notably, research on human colonic epithelial cells and mouse colon tissue confirms that HA fragments directly induce this Reg3 expression. Furthermore, cutaneous inflammation in mice induces a systemic inflammatory response, elevating mediators like IgE and TNF-α that aggravate intestinal inflammation [138,139]. This “reverse” axis signaling leads to a measurable decrease in total live bacteria in the mouse gut while simultaneously selecting for more virulent, opportunistic pathogens like *Bacteroides thetaiotaomicron*. These surviving microbes exhibit an enhanced capacity to penetrate the mucus layer and the colonic epithelium of the mice, thereby increasing the host’s susceptibility to severe colitis.

## 5. Therapeutic Perspectives

Therapeutic strategies that target the gut microbiota can reduce oxidative stress by promoting beneficial gut flora. The most common approaches include probiotics, prebiotics, synbiotics, and postbiotics, as well as fecal microbiota transplantation (FMT), while emphasizing the importance of a well-balanced diet and properly selected supplements [140].

### 5.1. Probiotics

According to the Food and Agriculture Organization (FAO) and the World Health Organization (WHO), probiotics are defined as live microorganisms that, when consumed in optimal amounts, confer a health benefit on the host [141,142]. The most commonly used probiotic species are Gram-positive bacteria, including *Lactobacillus* (*L. rhamnosus* GG, *L. sporogenes*, *L. reuteri* RC-14, *L. plantarum* 299v, *L. acidophilus*, *L. lactis*), *Bifidobacterium* spp. (*B. bifidum*, *B. longum*, *B. infantis*), *Streptococcus* spp. (*S. thermophillus*, *S. lactis*) and *Enterococcus* spp. These bacteria constitute part of the physiological intestinal flora in healthy individuals [143,144].

The primary role of probiotics is to maintain the balance of the intestinal microflora by competing with pathogenic bacteria for nutrients and adhesion sites on the intestinal epithelium, enhancing intestinal barrier function, modulating the immune response, and influencing other organs through the immune system and the production of neurotransmitters [145]. As mentioned earlier, probiotics reduce the severity of AD symptoms by increasing the production of Tregs, inhibiting the response of Th2 lymphocytes (type 2 T helper cells), improving the Th1/Th2 ratio, and reducing the secretion of pro-inflammatory cytokines (IL-4, IL-5, IL-6, IL-13, TNF-α, INF-γ) [146,147].

In addition, probiotics such as *Lactobacillus* and *Bifidobacterium* exhibit antioxidant activity by expressing enzymes like superoxide dismutase (SOD) and catalase (CAT), increasing glutathione (GSH) levels, and producing metabolites with antioxidant properties, such as butyrate, thereby limiting the formation of ROS [148,149,150]. It was observed that after supplementation with *L. casei* and *L. fermentum*, there was a significant increase in SOD and CAT [149,150]. Despite numerous studies, the exact mechanism of the antioxidant effect of probiotics is not yet fully understood.

A meta-analysis of 29 full-text articles, 15 of which were included in a pooled meta-analysis, evaluated the effect of probiotics on oxidative stress markers. The study demonstrated that probiotic supplementation at lower doses (less than 0.4 × 10^10^ CFU) resulted in a significant reduction in serum MDA concentrations. In addition, the probiotic supplementation resulted in increased levels of GSH, total antioxidant capacity (TAC), and nitric oxide (NO). Notably, the duration of probiotic supplementation significantly influences the modulation of oxidative stress. In studies with administration lasting less than 10 weeks, a decline in MDA levels, a marker of early-stage oxidative stress, was observed. This reduction may be attributable to the activation of SOD during the initial stages. In contrast, a protracted supplementation period (>10 weeks) facilitated the subsequent stages of antioxidant mechanisms by increasing GSH levels, which in turn are responsible for the neutralization of hydrogen peroxide (H_2_O_2_) generated by SOD [151]. The findings reported that the concurrent administration of probiotics with conventional AD treatment significantly enhanced the efficacy of the standard treatment in adults with the exogenous form of AD and the CC (C-159T) genotype [152].

Considering the high prevalence and impact of childhood AD, one meta-analysis suggests that administering *L. rhamnosus* to mothers and infants before and after birth may be an effective strategy for its prevention. However, further research is needed to determine the optimal dosage, duration of intervention, and underlying mechanisms of action [153]. Similarly, another review showed that supplementation with single- and multi-component probiotics reduces the risk of AD [154].

### 5.2. Prebiotics

Prebiotics are indigestible food components that specifically stimulate the growth or activity of advantageous bacterial strains in the intestines, thereby exerting a positive effect on the host’s health [141,155]. Prebiotics include dietary fiber (oligosaccharides), in particular fructooligosaccharides, galactooligosaccharides (GOSs), and inulin [156]. These substances have been shown to stimulate the growth of *Bifidobacterium* and *Lactobacillus*, primarily affecting the composition of the intestinal microflora [157]. Prebiotics undergo fermentation in the host’s digestive tract by the action of the intestinal microflora, leading to the production of SCFAs, namely acetate, propionate, and butyrate. These SCFAs exert anti-inflammatory effects, decrease the formation of toxic fermentation products, and stimulate nicotinamide adenine dinucleotide phosphate (NADPH) synthesis for GSH production, thereby reducing ROS generation [150,156]. Furthermore, prebiotics increase the Th1/Th2 ratio and the number of lymphocytes and/or leukocytes in gut-associated lymphoid tissues. They also enhance IgA (immunoglobulin A) secretion and have been shown to upregulate IL-18 (an anti-inflammatory cytokine) while downregulating IL-6 (an inflammatory biomarker) as well as genes involved in oxidative stress [156].

A study was conducted to investigate the effects of prebiotics, such as β-glucan and inulin, on the onset and progression of AD. To this end, the researchers employed an oxazolone (OX)-induced AD-like mouse model. Prebiotics were administered orally for 2 weeks after the end of the sensitization period (therapeutic study) and 3 weeks prior to its start (preventive study). The results of this study showed that prebiotic supplementation significantly reduced the severity of skin lesions and inflammatory responses. Furthermore, the dermis of mice treated with prebiotics exhibited a significant reduction in epidermal thickness and immune cell infiltration. Notably, early administration of β-glucan and inulin stimulated the growth of favorable gut bacteria in mice with AD, thereby preventing further AD advancement [132].

### 5.3. Synbiotics

The synergistic pairing of probiotics and prebiotics, known as synbiotics, has demonstrated high efficacy in product formulation [158]. Synbiotics are primarily designed to improve the survival and functionality of probiotic microorganisms by shielding them from gastric acid via prebiotics [141].

Administering a synbiotic composed of *Lactobacillus casei* and inulin may beneficially modulate oxidative stress markers such as MDA, H_2_O_2_, GSH, and the presence of free sulfhydryl groups [155]. Moreover, a meta-analysis confirmed the favorable impact of synbiotics on the management of AD in children aged 1 year or older [159].

### 5.4. Postbiotics

In addition to probiotics, prebiotics, and synbiotics, postbiotics are defined as bioactive metabolites or byproducts generated by probiotic microorganisms through fermentation processes [160]. Postbiotics naturally occur in products such as yogurt, sauerkraut, pickled vegetables, and kombucha, and are also produced by various bacterial and fungal species, including *Lactobacillus* spp., *Bifidobacterium* spp., *Streptococcus* spp., *Eubacterium* spp., *Faecalibacterium* spp., and *Saccharomyces* spp. [161]. Due to their unique structural properties, postbiotics provide storage stability and can activate diverse biological pathways involved in the modulation of inflammation and oxidative stress [162]. It should be emphasized that postbiotics, as non-viable microbial metabolites incapable of replication, represent a safer alternative to probiotics for individuals with impaired immune function [163].

Postbiotics derived from *Lactiplantibacillus plantarum* display antimicrobial effects against bacteria implicated in skin inflammation. They also modulate pro-inflammatory cytokines (IL-6, IL-8, TSLP) and genes related to the skin barrier (INV, FLG, LOR) in keratinocytes stimulated with TNF-α/IFN-γ, thereby strengthening the skin barrier [164]. One meta-analysis indicated that oral administration of *Lactobacillus* sp. postbiotics could potentially alleviate the severity of AD [165]. Despite promising findings, conclusive evidence for the clinical use of postbiotics in the treatment of AD is currently insufficient.

### 5.5. Fecal Microbiota Transplantation

FMT represents a promising therapeutic strategy for AD. It entails transferring intestinal microbiota from a healthy donor, obtained from a fecal sample, into the recipient’s gastrointestinal tract for therapeutic purposes [166]. FMT promotes an increase in butyric acid levels, leading to reduced LPS concentrations, attenuation of oxidative stress, inhibition of the inflammatory response, and preservation of intestinal barrier integrity [167]. In addition, FMT influences oxidative stress markers through the normalization of their expression [168].

Restoration of intestinal flora in mice with AD was investigated as a strategy to alleviate disease symptoms. Following FMT, the mice exhibited a reestablished Th1/Th2 balance, reduced IgE levels, and decreased numbers of mast cells, eosinophils, and basophils. By modulating the immune response, FMT may represent a novel approach for the treatment of AD in humans; however, further research is warranted [169,170]. Table 4 presents a summary of microbiota-targeted therapeutic strategies for AD.

### 5.6. Biomarkers and Targeted Therapeutic Strategies

Improved understanding of the pathophysiological mechanisms underlying AD has enabled the identification of disease-specific biomarkers, paving the way for the development of more effective and molecularly targeted therapeutic strategies, including anti-IL-31 monoclonal antibodies (nemolizumab), anti-OX40L agents (IMG-007), anti-TSLP therapies (APG777), and selective JAK1 inhibitors [176,177].

Nemolizumab is a humanized monoclonal antibody that acts as an antagonist of the α subunit of the interleukin-31 receptor, thereby inhibiting the IL-31 signaling pathway. Upon binding to its receptors in the skin, IL-31 promotes the recruitment of inflammatory cells, reduces the expression of epidermal barrier proteins, and activates sensory nerves, collectively contributing to pruritus [178,179]. Therefore, IL-31 represents a key therapeutic target in AD. Administration of nemolizumab has been shown to achieve a ≥75% reduction in skin lesions in 42–44% of patients after 16 weeks in phase III trials. Furthermore, concomitant use of nemolizumab with topical therapy resulted in a significant improvement in pruritus [177].

APG777 is a humanized monoclonal antibody that targets IL-13 via subcutaneous administration. It demonstrates substantial and sustained inhibition of key biomarkers in AD. Preliminary data suggest high efficacy and the potential for less frequent dosing (every 3–6 months) compared with current therapies. Phase-based clinical trials are currently ongoing [180]. IMG-007 is an innovative, long-acting anti-OX40 monoclonal antibody (anti-OX40 mAb) under investigation for the treatment of AD and alopecia areata. The antibody inhibits inflammatory pathways without eliminating T cells, potentially offering less frequent dosing (every 12 weeks) while maintaining a favorable safety profile [180]. These two drugs demonstrate considerable therapeutic and economic potential, as their extended dosing interval and prolonged duration of action reduce the burden on healthcare systems and are likely to improve patient adherence to treatment [180].

JAK inhibitors (JAKis) represent a novel class of therapeutics targeting dysregulated immune responses, including AD. JAKis have been shown to inhibit the intracellular JAK–STAT signaling pathway, thereby preventing the activation of multiple pro-inflammatory cytokines involved in disease development [181]. It is important to note that JAKis demonstrate high efficacy, but are also linked to comparatively higher risk of adverse effects [178]. The effectiveness and safety of topical ruxolitinib have been evaluated, leading to a reduction in inflammation and a faster antipruritic effect [182]. The clinical benefit of oral abrocitinib, administered at 100 mg or 200 mg in combination with dupilumab, has been evaluated in patients with moderate-to-severe AD requiring systemic treatment. Both medications exhibited comparable activity in reducing skin lesions, while the 200 mg dose of abrocitinib significantly alleviated the itching sensation after only 2 weeks of treatment [183]. Intervention with barticitinib, applied at doses of 2 mg or 4 mg, has been shown to result in sustained long-term therapeutic benefit in patients with moderate-to-severe AD, along with improvement in pruritus [184]. New targeted therapies, including JAKis and monoclonal antibodies, have demonstrated efficacy in clinical trials involving patients with moderate-to-severe AD. However, their precise mechanisms of action remain incompletely understood. Further clinical and experimental studies are required to elucidate these molecular mechanisms and to assess long-term safety.

## 6. The Role of Antioxidants and Antioxidant Supplementation in AD

This article outlines the mechanisms of oxidative stress, which play a central role in the pathogenesis of AD by damaging the epidermal barrier and sustaining chronic inflammation. Consequently, it is reasonable to consider antioxidants, whose primary function is to neutralize ROS and thereby restore oxidative–antioxidant equilibrium [185,186]. The most extensively studied antioxidants in AD include vitamins D (VD), E (VE), and C (VC), carotenoids, and melatonin [187]. A meta-analysis suggests that antioxidant therapy may significantly reduce the severity of AD, but has no notable effect on alleviating itching. The study included 18 randomized clinical trials involving 763 patients with AD. The greatest therapeutic benefit was observed with oral supplementation of vitamin D, combined VD and VE supplementation, a combination of vitamins A (VA), VD, and VE, and topical vitamin B12. Overall, antioxidants may represent a safe and potentially effective adjunctive treatment for patients with AD [188].

### 6.1. Vitamin D

VD is a group of fat-soluble steroid compounds produced endogenously in the skin upon exposure to ultraviolet (UV) radiation from sunlight, which triggers its synthesis [189]. It is evident that VD exerts a significant influence on the process of keratinocyte differentiation, as well as on the production of antimicrobial peptides, including cathelicidin and human beta-defensin. These peptides have been shown to contribute to the reinforcement of the skin barrier by promoting filaggrin synthesis and inhibiting the production of inflammatory cytokines. In addition, VD plays a pivotal role in the prevention of skin infections [187,190]. VD modulates Th2/Th17 pathways, supporting Treg activity and thereby potentially ameliorating AD symptoms [190]. A study was conducted to evaluate changes in the severity of AD during oral VD supplementation at 1000 IU/day (25 μg/day) in 39 children with AD and 20 healthy, non-allergic children over a period of 3 months. Following supplementation, reductions were observed in both the SCORAD index and levels of pro-inflammatory cytokines, including IL-2, IL-4, IL-6, and IFN-γ [191]. Another study reported that, in addition to a reduction in SCORAD, there was also an increase in the expression of VD receptors and cathelicidin in affected skin. Cathelicidin is a protein belonging to the family of AMPs with antimicrobial properties [192]. A meta-analysis of 11 randomized controlled trials involving 686 patients with AD confirmed that VD supplementation mitigated the intensity of AD in both children and adults [193]. Additionally, it was observed that AD patients with lower serum VD concentrations experienced a more severe course of the disease [194]. Following a comprehensive analysis of the extant literature, it is concluded that the positive effects of VD supplementation and the alleviation of AD symptoms require confirmation through larger-scale studies involving longer treatment periods.

### 6.2. Vitamin E

VE (tocopherols and tocotrienols) is a fat-soluble, potent biological antioxidant that protects cells from oxidative stress by neutralizing oxygen free radicals and promoting keratinocyte differentiation [36]. A meta-analysis suggests that the administration of VE at a dose of 400 IU per day may improve itching sensation in patients with AD [195]. An investigation aimed to evaluate the relationship between higher VE supplementation and total IgE/specific IgE levels in serum in children with AD. The study proposed that relatively higher VE intake may lower total serum IgE levels, thereby improving AD symptoms [196].

### 6.3. Vitamin C

VC (also known as L-ascorbic acid) is an essential water-soluble antioxidant that protects cells from oxidative stress, participates in collagen biosynthesis as well as carnitine and catecholamine metabolism, supports immune function, and enhances dietary iron absorption. It is obtained exclusively from the diet through the consumption of fruits and vegetables [197]. VC helps maintain the integrity of the epidermis by preventing water loss in the skin [198]. An open-label study involving 20 patients with AD investigated the administration of a preparation containing poly-L-lysine in combination with VA and VC for a period of 28 days. The study observed a 41.8% reduction in disease intensity on the SCORAD scale and improvement in quality of life, as well as a decrease in erythema, pruritus, and dryness [199]. Another investigation aimed to assess the impact of a maternal diet rich in VC and VE and its potential protective effect against the development of atopy in newborns. It was reported that higher concentrations of VC in breast milk correlate with a reduced risk of atopy in infants [200]. However, as the study was conducted on a small group, it is difficult to state unequivocally that VC reduces the severity of AD symptoms. Further studies are needed to confirm its effectiveness.

### 6.4. Carotenoids

Carotenoids (including β-carotene, α-carotene, γ-carotene, and β-cryptoxanthin) are natural, hydrophobic pigments synthesized by plants, algae, and bacteria that give plants their yellow, orange, and red colors. They act as antioxidants, protecting the photosynthetic apparatus from photooxidation, and also serve as precursors of VA. Carotenoids play a role in modulating the immune system, cell communication, embryonic development, hematopoiesis, and apoptosis. These pigments also exhibit antioxidant, anti-inflammatory, anti-angiogenic, and anti-proliferative properties [201]. Carotenoids reduce ROS levels, thereby decreasing lipid peroxidation and increasing the activity of endogenous antioxidant enzymes such as superoxide dismutase and glutathione peroxidase. This action is particularly relevant to the protection of the skin barrier [202,203]. Additionally, carotenoids have been reported to reduce the expression of pro-inflammatory cytokines such as TNF-α, IL-6, and IL-1β, and to inhibit COX-2 (cyclooxygenase-2) and LOX (lysyl oxidase), thereby exerting anti-inflammatory effects on the skin [203]. A study showed that consuming more green and yellow vegetables, citrus fruits, and β-carotene during pregnancy may help prevent eczema and asthma in children [204]. Another study suggested that higher concentrations of lutein, a yellow pigment belonging to the carotenoid family, were significantly associated with a lower risk of AD [205].

## 7. Conclusions

AD is a common inflammatory skin disease affecting both children and adults worldwide. Despite its prevalence, the pathophysiology of AD remains an elusive subject of scientific inquiry and continues to serve as a focal point in the search for novel therapeutic strategies. Among the main predisposing factors for the development of AD are skin barrier dysfunction, dysbiosis of the skin and gut microbiota, genetic factors (most notably filaggrin gene mutations), environmental influences, and dysregulation of the immune response. The presented review highlights that the interplay between gut dysbiosis and oxidative stress represents a central pathogenic axis in atopic dermatitis. Dysbiosis diminishes the production of microbial-derived metabolites with immunoregulatory and antioxidant properties, leading to enhanced inflammatory responses and increased ROS generation. Elevated ROS levels, in turn, further damage the microbiota and epithelial barrier, perpetuating dysbiosis and allowing systemic dissemination of microbial and inflammatory mediators that ultimately affect the skin. These interconnected processes establish a chronic, self-reinforcing feedback loop that sustains inflammation and exacerbates disease. Moreover, emerging therapeutic approaches involving the use of microorganisms—such as probiotics, prebiotics, synbiotics, postbiotics, and fecal microbiota transplantation (FMT)—offer promising perspectives for restoring microbial balance and mitigating disease severity. Importantly, future research should aim to elucidate more precisely the complex interactions between gut microbiota composition, oxidative stress, and immune regulation in atopic dermatitis.

## 8. Future Directions

Future studies should focus on well-designed randomized controlled trials with larger sample sizes that clarify the exact mechanisms involved in the interaction between gut microbiota and oxidative stress in AD. It is particularly important to consider the timing of supplementation (prenatal, postnatal, or both) and to identify the strains and doses that provide the greatest benefits to patients. In addition, it is worth emphasizing the need to assess the safety and durability of the therapeutic effects obtained.

## Figures and Tables

**Table 1:** Skin dysbiosis associated with atopic dermatitis.

Dermatosis	Excessive Microbial Populations	Reduced Microbial Populations	References
AD	*S. aureus*	*C. acnes*	[60]
	*S. aureus*, *C. tuberculostearicum*, *S. capitis*, *S. lugdunensis*, *S. epidermidis*, *Candida* spp.	*Malassezia* spp.	[65]
	*S. aureus*, *S. haemolyticus*, *S. capitis*, *S.lugdunensis*, *S. epidermidis*, *C. albicans*, *Aspergillus* spp., *C. diffluens*	*Corynebacterium* spp., *Acinetobacter* spp., *Cutibacterium* spp., *Prevotella* spp., *Malassezia* spp., *Streptococcus* spp.	[67]
	*S. aureus*	*Streptococcus* spp., *Cutibacterium* spp., *Corynebacterium* spp.	[69]
	*S. aureus*	*Cutibacterium* spp., *Corynebacterium* spp., *Streptococcus* spp.	[72]

Abbreviations: AD—atopic dermatitis; *C. acnes*—*Cutibacterium acnes*; *C. albicans*—*Candida albicans*; *C. diffluens*—*Cryptococcus diffluens*; *C. tuberculostearicum*—*Corynebacterium tuberculostearicum*; *S. aureus*—*Staphylococcus aureus*; *S. capitis*—*Staphylococcus capitis*; *S. epidermidis*—*Staphylococcus epidermidis*; *S. haemolyticus*—*Staphylococcus haemolyticus*; *S. lugdunensis*—*Staphylococcus lugdunensis*.

**Table 2:** Bacteria with potential protective effects on AD development.

Bacteria	Protective Effects	Reference
*Clostridia* UCG-014	Decreasing MDC/CCL22 levels	[106]
*Clostridiales* Family XIII Incertae Sedis	Producing SCFAs	[106]
*Akkermansia* genus	Producing SCFAs	[116]
*Bifidobacteria* genus	Producing SCFAs, γ-aminobutyric acid	[105,110,116]
*F. prausnitzii*	Producing SCFAs	[116,123]
*Eubacteriaceae* family, e.g., *E. coprostanoligenes*	Producing SCFAs—especially butyrate—lowering the levels of the pro-inflammatory eotaxin/CCL11, MDC/CCL22, and Flt3L	[106]
*Lactobacillus* genus	Producing γ-aminobutyric acid	[105]
*B. longum*, *B. bifidum*	Digesting HMO in breastmilk and producing beneficial aromatic lactic acids	[107]
*Veillonellaceae* family	Mediating circulating inflammatory cytokine IL-15RA	[109]
*Fusicatenibacter* genus	Regulating IL-10 production and Treg differentiation	[109]
*Odoribacter* genus	Regulating IL-10 production and Treg differentiation	[109]
*Barnesiellaceae* family	Decreasing MDC/CCL22 levels	[106]
*Oscillospiraceae* family	Decreasing MDC/CCL22 levels	[106]
*Peptococcaceae* family	Decreasing MDC/CCL22 levels	[106]

Abbreviations: *B. bifidum*—*Bifidobacterium bifidum*; *B. longum*—*Bifidobacterium longum*; *E. coprostanoligenes*—*Eubacterium coprostanoligenes*; *F. prausnitzii*—*Faecalibacterium prausnitzii*; eotaxin/CCL11—eosinophil chemotactic protein/C-C motif chemokine 11; Flt3L—fms-related tyrosine kinase 3 ligand; HMO—human milk oligosaccharide; IL-10—interleukin 10; IL-15RA—interleukin 15 receptor alpha subunit; MDC/CCL22—macrophage-derived chemokine/C-C motif chemokine 22; SCFAs—short-chain fatty acids; Treg—regulatory T lymphocyte.

**Table 3:** Bacteria with potential adverse effects on AD development.

Bacteria	Adverse Effects	Reference
*Clostridium* genus	Triggering eosinophilic inflammation, excessive toxin release, inhibiting the chemotaxis of neutrophils and inactive eosinophils	[115,116]
*C. difficile*	Contributing to intestinal epithelial barrier leak	[117]
*E. coli*	Contributing to intestinal epithelial barrier leak	[117]
*B. adolescentis*, *B. catenulatum*	Premature shift towards adult-type microbiota	[107,108,121]
*Pasteurellaceae* family	Increasing MDC/CCL22 levels	[106]
*Lachnospiraceae* UCG001	Deteriorating amino acid metabolism, increasing blood uric acid levels and contributing to CD4 TH17-driven inflammation	[116]

Abbreviations: *B. adolescentis*—*Bifidobacterium adolescentis*; *B. catenulatum*—*Bifidobacterium catenulatum*; *C. difficile*—*Clostridoides difficile*; CD4 TH17—T helper 17 lymphocyte; *E. coli*—*Escherichia coli*; MDC/CCL22—macrophage-derived chemokine/C-C motif chemokine 22.

**Table 4:** Microbiota-targeted therapeutic strategies for the treatment of AD.

Therapeutic Approach	Mechanism of Action	Example	Documented or Potential Effects	Limitations	Reference
Probiotics	Modulation of intestinal microflora; strengthening of the intestinal barrier; immunomodulation; reduction in Th2-dependent inflammation	*L. rhamnosus GG*, *B.longum*, *L. paracasei*	Reduction in pro-inflammatory cytokine levels (IL-4 and IgE); improvement in skin barrier function	Variability of action depending on strain and dose	[132,156,171]
Prebiotics	Stimulation of the growth of beneficial bacteria that produce SCFA	GOS, inulin, FOS	Reduction in skin symptoms; improvement in immune response through increased IL-18	Lack of standardization of preparation, limited number of large clinical trials confirming efficacy	[150,156,172]
Postbiotics	Probiotic metabolites; strengthening of the intestinal barrier	Butyric acid, bacteriocins, lipoteichoic acid from *L. plantarum*	Strengthening the epithelial barrier through modulation of pro-inflammatory cytokines (IL-6, IL-8)	Lack of standardization and regulation of doses	[162,164,173]
Synbiotics	Combination of probiotic and prebiotic, synergistic improvement inmicrobiota and modulation of oxidative stress	*L. rhamnosus* GG and GOS	More effectively relieve skin symptoms	Heterogeneity of preparations and compositions; lack of optimal doses and duration of therapy	[158,159,174]
FMT	Restoration of intestinal flora; modulation of the immune system	FMT from healthy donors (preliminary studies)	Relief of skin inflammation	Risk of pathogen transmission; donor diversity	[166,167,175]

Abbreviations: *B. longum*—*Bifidobacterium longum*; FMT—fecal microbiota transplantation; FOS—fructooligosaccharide; GOS—galactooligosaccharide; *L. paracasei*—*Lactobacillus paracasei*; *L. plantarum*—*Lactobacillus plantarum*; *L. rhamnosus*—*Lactobacillus rhamnosus*; SCFA—short-chain fatty acid.

## Data Availability

Data are contained within the article.

## Abbreviations

The following abbreviations are used in this manuscript: 8-OHdG—8-hydroxy-2′-deoxyguanosine; AD—atopic dermatitis; AhR—aryl hydrocarbon receptor; AMPs—antimicrobial peptides; *B. catenulatum*—*Bifidobacterium catenulatum*; *B. longum*—*Bifidobacterium longum*; B cell—B lymphocyte; CAT—catalase; CD4 TH17—T helper 17 lymphocyte; *C. difficile*—*Clostridoides difficile*; COX-2—cyclooxygenase-2; DC—dendritic cell; DCs—dendritic cells; eotaxin/CCL11—eosinophil chemotactic protein/C-C motif chemokine 11; *E. coli*—*Escherichia coli*; FAO—Food and Agriculture Organization; FLG—filaggrin; Flt3L—fms-related tyrosine kinase 3 ligand; FMT—fecal microbiota transplantation; FOS—fructooligosaccharide; *F. prausnitzii*—*Faecalibacterium prausnitzii*; GOS—galactooligosaccharide; GSH—glutathione; H2O2—hydrogen peroxide; HMO—human milk oligosaccharide; IFN-γ—interferon gamma; IgA—immunoglobulin A; IgE—immunoglobulin E; IL-1—interleukin 1; IL-1β—interleukin 1 beta; IL-2—interleukin 2; IL-4—interleukin 4; IL-5—interleukin 5; IL-6—interleukin 6; IL-8—interleukin 8; IL-10—interleukin 10; IL-13—interleukin 13; IL-15RA—interleukin 15 receptor alpha subunit; IL-17—interleukin 17; IL-18—interleukin 18; IL-25—interleukin 25; IL-31—interleukin 31; IL-33—interleukin 33; KGH—keratohyalin granule; LC—Langerhans cell; LCs—Langerhans cells; LOX—lysyl oxidase; LPS—lipopolysaccharide; *L. rhamnosus*—*Lactobacillus rhamnosus*; MDA—malondialdehyde; MC—mast cell; MDC/CCL22—macrophage-derived chemokine/C-C motif chemokine 22; MIP-1α/CCL3—macrophage inflammatory protein 1-α/chemokine (C-C motif) ligand 3; NMF—natural moisturizing factor; NADPH—nicotinamide adenine dinucleotide phosphate; NO—nitric oxide; NOD—nucleotide-binding oligomerization domain-like receptor; PAMPs—pathogen-associated molecular patterns; PCA—pyrrolidone carboxylic acid; ROS—reactive oxygen species; *S. aureus*—*Staphylococcus aureus*; SC—stratum corneum; SCFAs—short-chain fatty acids; SCORAD—SCORing Atopic Dermatitis; SOD—superoxide dismutase; TAC—total antioxidant capacity; TEWL—transepidermal water loss; Th1—T helper 1 lymphocyte; Th2—T helper 2 lymphocyte; Th17—T helper 17 lymphocyte; Th22—T helper 22 lymphocyte; Treg—regulatory T lymphocyte; Tregs—regulatory T lymphocytes; TLRs—toll-like receptors; TNF—tumor necrosis factor; TNF-α—tumor necrosis factor-alpha; TSLP—thymic stromal lymphopoietin; UCA—urocanic acid; VEGF—vascular endothelial growth factor; WHO—World Health Organization.

## References

[1] Rowland I., Gibson G., Heinken A., Scott K., Swann J., Thiele I., Tuohy K. (2018). Gut Microbiota Functions: Metabolism of Nutrients and Other Food Components. Eur. J. Nutr..

[2] Morrison D.J., Preston T. (2016). Formation of Short Chain Fatty Acids by the Gut Microbiota and Their Impact on Human Metabolism. Gut Microbes.

[3] Mohammad I., Ansari M.R., Khan M.S., Bari M.N., Kamal M.A., Poyil M.M. (2025). Beyond Digestion: The Gut Microbiota as an Immune–Metabolic Interface in Disease Modulation. Gastrointest. Disord..

[4] Qu S., Yu Z., Zhou Y., Wang S., Jia M., Chen T., Zhang X. (2024). Gut Microbiota Modulates Neurotransmitter and Gut-Brain Signaling. Microbiol. Res..

[5] Strandwitz P. (2018). Neurotransmitter Modulation by the Gut Microbiota. Brain Res..

[6] Fu Y., Lyu J., Wang S. (2023). The Role of Intestinal Microbes on Intestinal Barrier Function and Host Immunity from a Metabolite Perspective. Front. Immunol..

[7] Neurath M.F., Artis D., Becker C. (2025). The Intestinal Barrier: A Pivotal Role in Health, Inflammation, and Cancer. Lancet Gastroenterol. Hepatol..

[8] Yurtseven B., Aydemir E., Ayaz F. (2025). The Role of Intestinal Microbiota and Immune System Interactions in Autoimmune Diseases. ImmunoTargets Ther..

[9] Molloy M., Bouladoux N., Belkaid Y. (2012). Intestinal Microbiota: Shaping Local and Systemic Immune Responses. Semin. Immunol..

[10] Makki K., Deehan E.C., Walter J., Bäckhed F. (2018). The Impact of Dietary Fiber on Gut Microbiota in Host Health and Disease. Cell Host Microbe.

[11] Penders J., Thijs C., Vink C., Stelma F.F., Snijders B., Kummeling I., Van Den Brandt P.A., Stobberingh E.E. (2006). Factors Influencing the Composition of the Intestinal Microbiota in Early Infancy. Pediatrics.

[12] Russell S.L., Gold M.J., Hartmann M., Willing B.P., Thorson L., Wlodarska M., Gill N., Blanchet M.R., Mohn W.W., McNagny K.M. (2012). Early Life Antibiotic-Driven Changes in Microbiota Enhance Susceptibility to Allergic Asthma. EMBO Rep..

[13] Karl J.P., Margolis L.M., Madslien E.H., Murphy N.E., Castellani J.W., Gundersen Y., Hoke A.V., Levangie M.W., Kumar R., Chakraborty N. (2017). Changes in Intestinal Microbiota Composition and Metabolism Coincide with Increased Intestinal Permeability in Young Adults under Prolonged Physiological Stress. Am. J. Physiol. Gastrointest. Liver Physiol..

[14] Garcia I., Kilic F., Bryan C.A., Castro-Vildosola J., Jonnalagadda S.A., Kasturi A., Tilly J., Smith J., Valentin S., Moncayo S. (2025). Social Stress Changes Gut Microbiome Composition in Male, Female, and Aggressor Mice. Brain Behav. Immun. Health.

[15] Claus S.P., Guillou H., Ellero-Simatos S. (2016). The Gut Microbiota: A Major Player in the Toxicity of Environmental Pollutants?. npj Biofilms Microbiomes.

[16] Barra N.G., Fang H., Bhatwa A., Schmidt A.M., Syed S.A., Steinberg G.R., Morrison K.M., Surette M.G., Wade M.G., Holloway A.C. (2025). Food Supply Toxicants and Additives Alter the Gut Microbiota and Risk of Metabolic Disease. Am. J. Physiol. Endocrinol. Metab..

[17] Xue R., Zong X., Jiang X., You G., Wei Y., Guo B. (2026). Artificial Intelligence-Driven Food Safety: Decoding Gut Microbiota-Mediated Health Effects of Non-Microbial Contaminants. Foods.

[18] Kunst C., Schmid S., Michalski M., Tümen D., Buttenschön J., Müller M., Gülow K. (2023). The Influence of Gut Microbiota on Oxidative Stress and the Immune System. Biomedicines.

[19] Shandilya S., Kumar S., Kumar Jha N., Kumar Kesari K., Ruokolainen J. (2022). Interplay of Gut Microbiota and Oxidative Stress: Perspective on Neurodegeneration and Neuroprotection. J. Adv. Res..

[20] Ashrafi F., Advani S., Pinto-Tomás A.A., Jeste D.V. (2025). Oxidative Stress-Microbiota-Epigenetics Crosstalk: A Missing Link Between Cognition and Social Behavior in Metabolic and Neuropsychiatric Disorders. Cells.

[21] Das A., De A.J., Mohanty T., Aich P. (2023). Role of Oxidative Stress, Gut Microbiota and Derived Metabolites in the Etiology and Progression of Nonalcoholic Fatty Liver Disease. Redox Exp. Med..

[22] Pan Y., Cheng J.H., Sun D.W. (2021). Metabolomic Analyses on Microbial Primary and Secondary Oxidative Stress Responses. Compr. Rev. Food Sci. Food Saf..

[23] Zhao M., Chu J., Feng S., Guo C., Xue B., He K., Li L. (2023). Immunological Mechanisms of Inflammatory Diseases Caused by Gut Microbiota Dysbiosis: A Review. Biomed. Pharmacother..

[24] Malicevic U., Rai V., Skrbic R., Agrawal D.K. (2024). NLRP3 Inflammasome and Gut Dysbiosis Linking Diabetes Mellitus and Inflammatory Bowel Disease. Arch. Intern. Med. Res..

[25] Chen J., Jia S., Xue X., Guo C., Dong K. (2024). Gut Microbiota: A Novel Target for Exercise-Mediated Regulation of NLRP3 Inflammasome Activation. Front. Microbiol..

[26] Nermes M., Kantele J.M., Atosuo T.J., Salminen S., Isolauri E. (2011). Interaction of Orally Administered *Lactobacillus rhamnosus* GG with Skin and Gut Microbiota and Humoral Immunity in Infants with Atopic Dermatitis. Clin. Exp. Allergy.

[27] Wollenberg A., Barbarot S., Bieber T., Christen-Zaech S., Deleuran M., Fink-Wagner A., Gieler U., Girolomoni G., Lau S., Muraro A. (2018). Consensus-Based European Guidelines for Treatment of Atopic Eczema (Atopic Dermatitis) in Adults and Children: Part I. J. Eur. Acad. Dermatol. Venereol..

[28] Mao Q., Wang X., Cai H., Yang J., Zhang Y., Min W., Qian Q., Zeng Y. (2024). Research Progress on the Correlation of Atopic Dermatitis with Gut Microbiota. Clin. Cosmet. Investig. Dermatol..

[29] Luo Y., Huang X., Hu H., Wang Y., Feng X., Chen S., Luo H. (2024). Intestinal Microflora Promotes Th_2_-Mediated Immunity through NLRP3 in Damp and Heat Environments. Front. Immunol..

[30] Yu B., Dai C.Q., Chen J., Deng L., Wu X.L., Wu S., Zhao C.L., Jiang Z.Y., Chen X.Y. (2015). Dysbiosis of Gut Microbiota Induced the Disorder of Helper T Cells in Influenza Virus-Infected Mice. Hum. Vaccines Immunother..

[31] Grewe W., Bruijnzeel-Koomen C.A., Schöpf E., Thepen T., Langeveld-Wildschut A.G., Ruzicka T., Krutmann J. (1997). A Role for IL-12 in Regulation of Atopic Dermatitis. Immunol. Today.

[32] Remigante A., Morabito R. (2024). Oxidative Stress and Antioxidant Strategies: Relationships and Cellular Pathways for Human Health. Cells.

[33] Liu H., Jiao Y., Wang P.-C., Chen Y., Xu M., Zhang X., Zheng X., Yang Z. (2026). Oxidative Stress and Antioxidant Therapeutic Mechanisms. Pharmacol. Ther..

[34] Sendtner N., Seitz R., Brandl N., Müller M., Gülow K. (2025). Reactive Oxygen Species Across Death Pathways: Gatekeepers of Apoptosis, Ferroptosis, Pyroptosis, Paraptosis, and Beyond. Int. J. Mol. Sci..

[35] Manoharan R.R., Prasad A., Pospíšil P., Kzhyshkowska J. (2024). ROS Signaling in Innate Immunity via Oxidative Protein Modifications. Front. Immunol..

[36] Kvedariene V., Vaskovic M., Semyte J.B. (2025). Role of Oxidative Stress and Antioxidants in the Course of Atopic Dermatitis. Int. J. Mol. Sci..

[37] Luo Y., Hu J., Zhou Z., Zhang Y., Wu Y., Sun J. (2025). Oxidative Stress Products and Managements in Atopic Dermatitis. Front. Med..

[38] Tsukahara H., Shibata R., Ohshima Y., Todoroki Y., Sato S., Ohta N., Hiraoka M., Yoshida A., Nishima S., Mayumi M. (2003). Oxidative Stress and Altered Antioxidant Defenses in Children with Acute Exacerbation of Atopic Dermatitis. Life Sci..

[39] Ravnborg N., Ambikaibalan D., Agnihotri G., Price S., Rastogi S., Patel K.R., Singam V., Andersen Y., Halling A.S., Silverberg J.I. (2021). Prevalence of Asthma in Patients with Atopic Dermatitis: A Systematic Review and Meta-Analysis. J. Am. Acad. Dermatol..

[40] Steiner U.C., Bachmann L.M., Soyka M.B., Regenass S., Steinegger L., Probst E. (2018). Relationship Between Rhinitis, Asthma, and Eczema and the Presence of Sensitization in Young Swiss Adults. Allergy Rhinol..

[41] Knudgaard M.H., Andreasen T.H., Ravnborg N., Bieber T., Silverberg J.I., Egeberg A., Halling A.-S., Thyssen J.P. (2021). Rhinitis Prevalence and Association with Atopic Dermatitis. A Systematic Review and Meta-Analysis. Ann. Allergy Asthma Immunol..

[42] Boothe W.D., Tarbox J.A., Tarbox M.B. (2024). Atopic Dermatitis: Pathophysiology. Adv. Exp. Med. Biol..

[43] Eichenfield L.F., Stripling S., Fung S., Cha A., O’Brien A., Schachner L.A. (2022). Recent Developments and Advances in Atopic Dermatitis: A Focus on Epidemiology, Pathophysiology, and Treatment in the Pediatric Setting. Paediatr. Drugs.

[44] Langan S.M., Irvine A.D., Weidinger S. (2020). Atopic Dermatitis. Lancet.

[45] Schuler C.F., Tsoi L.C., Billi A.C., Harms P.W., Weidinger S., Gudjonsson J.E. (2023). Genetic and Immunological Pathogenesis of Atopic Dermatitis. J. Investig. Dermatol..

[46] Moosbrugger-Martinz V., Leprince C., Méchin M.C., Simon M., Blunder S., Gruber R., Dubrac S. (2022). Revisiting the Roles of Filaggrin in Atopic Dermatitis. Int. J. Mol. Sci..

[47] Drislane C., Irvine A.D. (2020). The Role of Filaggrin in Atopic Dermatitis and Allergic Disease. Ann. Allergy Asthma Immunol..

[48] Cabanillas B., Novak N. (2016). Atopic Dermatitis and Filaggrin. Curr. Opin. Immunol..

[49] Stefanovic N., Irvine A.D. (2024). Filaggrin and beyond: New Insights into the Skin Barrier in Atopic Dermatitis and Allergic Diseases, from Genetics to Therapeutic Perspectives. Ann. Allergy Asthma Immunol..

[50] Freeman S.C., Sonthalia S. (2023). Histology, Keratohyalin Granules. StatPearls.

[51] Irvine A.D., McLean W.H.I. (2006). Breaking the (Un)Sound Barrier: Filaggrin Is a Major Gene for Atopic Dermatitis. J. Investig. Dermatol..

[52] Yang G., Seok J.K., Kang H.C., Cho Y.Y., Lee H.S., Lee J.Y. (2020). Skin Barrier Abnormalities and Immune Dysfunction in Atopic Dermatitis. Int. J. Mol. Sci..

[53] Mu Z., Zhao Y., Liu X., Chang C., Zhang J. (2014). Molecular Biology of Atopic Dermatitis. Clin. Rev. Allergy Immunol..

[54] Howell M.D., Kim B.E., Gao P., Grant A.V., Boguniewicz M., DeBenedetto A., Schneider L., Beck L.A., Barnes K.C., Leung D.Y.M. (2007). Cytokine Modulation of Atopic Dermatitis Filaggrin Skin Expression. J. Allergy Clin. Immunol..

[55] Thyssen J.P., Kezic S. (2014). Causes of Epidermal Filaggrin Reduction and Their Role in the Pathogenesis of Atopic Dermatitis. J. Allergy Clin. Immunol..

[56] Goleva E., Berdyshev E., Leung D.Y.M. (2019). Epithelial Barrier Repair and Prevention of Allergy. J. Clin. Investig..

[57] Kezic S., O’Regan G.M., Yau N., Sandilands A., Chen H., Campbell L.E., Kroboth K., Watson R., Rowland M., Irwin McLean W.H. (2011). Levels of Filaggrin Degradation Products Are Influenced by Both Filaggrin Genotype and Atopic Dermatitis Severity. Allergy.

[58] Kezic S., Kemperman P.M.J.H., Koster E.S., De Jongh C.M., Thio H.B., Campbell L.E., Irvine A.D., McLean I.W.H., Puppels G.J., Caspers P.J. (2008). Loss-of-Function Mutations in the Filaggrin Gene Lead to Reduced Level of Natural Moisturizing Factor in the Stratum Corneum. J. Investig. Dermatol..

[59] Andrew P.V., Williams S.F., Brown K., Chittock J., Pinnock A., Poyner A., Cork M.J., Danby S.G. (2025). Topical Supplementation with Physiological Lipids Rebalances the Stratum Corneum Ceramide Profile and Strengthens Skin Barrier Function in Adults Predisposed to Atopic Dermatitis. Br. J. Dermatol..

[60] Rozas M., de Ruijter A.H., Fabrega M.J., Zorgani A., Guell M., Paetzold B., Brillet F. (2021). From Dysbiosis to Healthy Skin: Major Contributions of Cutibacterium Acnes to Skin Homeostasis. Microorganisms.

[61] Grice E.A. (2014). The Skin Microbiome: Potential for Novel Diagnostic and Therapeutic Approaches to Cutaneous Disease. Semin. Cutan. Med. Surg..

[62] Grice E.A., Segre J.A. (2011). The Skin Microbiome. Nat. Rev. Microbiol..

[63] Chen Y., Knight R., Gallo R.L. (2023). Evolving Approaches to Profiling the Microbiome in Skin Disease. Front. Immunol..

[64] Gallo R.L. (2017). Human Skin Is the Largest Epithelial Surface for Interaction with Microbes. J. Investig. Dermatol..

[65] Hülpüsch C., Rohayem R., Reiger M., Traidl-Hoffmann C. (2024). Exploring the Skin Microbiome in Atopic Dermatitis Pathogenesis and Disease Modification. J. Allergy Clin. Immunol..

[66] Han J.H., Kim H.S. (2024). Skin Deep: The Potential of Microbiome Cosmetics. J. Microbiol..

[67] Baglama Š.Š., Trčko K. (2022). Skin and Gut Microbiota Dysbiosis in Autoimmune and Inflammatory Skin Diseases. Acta Dermatovenerol. Alp. Pannonica Adriat..

[68] Grobe W., Bieber T., Novak N. (2019). Pathophysiology of Atopic Dermatitis. JDDG J. Ger. Soc. Dermatol..

[69] Ferček I., Ozretić P., Zanze L., Zoričić Z., Dolački L., Čivljak R., Lugović-Mihić L. (2025). The Role of Skin Microbiota in Facial Dermatoses and Related Factors: A Narrative Review. Int. J. Mol. Sci..

[70] Peng G., Abudouwanli A., Sun Q., Tan Y., Zhao W., Yang M., Wang S., Ogawa H., Okumura K., Niyonsaba F. (2025). Role of Antimicrobial Peptides in the Pathogenesis of Atopic Dermatitis. J. Dermatol..

[71] Gatmaitan J.G., Lee J.H. (2023). Challenges and Future Trends in Atopic Dermatitis. Int. J. Mol. Sci..

[72] Boggio C.M.T., Veronese F., Armari M., Zavattaro E., Esposto E., Savoia P., Azzimonti B. (2025). Skin Microbiota in Atopic Dermatitis: Victim or Executioner?. Clin. Microbiol. Rev..

[73] Schmuth M., Eckmann S., Moosbrugger-Martinz V., Ortner-Tobider D., Blunder S., Trafoier T., Gruber R., Elias P.M. (2024). Skin Barrier in Atopic Dermatitis. J. Investig. Dermatol..

[74] Egawa G., Kabashima K. (2018). Barrier Dysfunction in the Skin Allergy. Allergol. Int..

[75] Tsukui K., Suzuki M., Amma M., Tokudome Y. (2024). Synergistic Effect of Cerium Chloride and Calcium Chloride Alters Calcium Signaling in Keratinocytes to Promote Epidermal Differentiation. Biosci. Biotechnol. Biochem..

[76] Luger T., Amagai M., Dreno B., Dagnelie M.A., Liao W., Kabashima K., Schikowski T., Proksch E., Elias P.M., Simon M. (2021). Atopic Dermatitis: Role of the Skin Barrier, Environment, Microbiome, and Therapeutic Agents. J. Dermatol. Sci..

[77] Afshari M., Kolackova M., Rosecka M., Čelakovská J., Krejsek J. (2024). Unraveling the Skin; a Comprehensive Review of Atopic Dermatitis, Current Understanding, and Approaches. Front. Immunol..

[78] Lopez D.J., Lodge C.J., Bui D.S., Waidyatillake N.T., Su J.C., Perret J.L., Knibbs L.D., Erbas B., Thomas P.S., Hamilton G.S. (2021). Association between Ambient Air Pollution and Development and Persistence of Atopic and Non-Atopic Eczema in a Cohort of Adults. Allergy Eur. J. Allergy Clin. Immunol..

[79] Park S.K., Kim J.S., Seo H.M. (2022). Exposure to Air Pollution and Incidence of Atopic Dermatitis in the General Population: A National Population-Based Retrospective Cohort Study. J. Am. Acad. Dermatol..

[80] Kim B.E., Kim J., Goleva E., Berdyshev E., Lee J., Vang K.A., Lee U.H., Han S.Y., Leung S., Hall C.F. (2021). Particulate Matter Causes Skin Barrier Dysfunction. JCI Insight.

[81] Wang Z., Zhang M. (2023). Smoking and the Risk of Atopic Dermatitis: A Two-Sample Mendelian Randomization Study. Medicine.

[82] Tu J., Wan W., Tang B., Jiang F., Wen J., Luo Q., Ye J. (2024). Dissecting the Pathogenic Effects of Smoking in Blood DNA Methylation on Allergic Diseases. World Allergy Organ. J..

[83] Yoshida S., Mishina H., Takeuchi M., Kawakami K. (2021). Association of Prenatal Maternal, Prenatal Secondhand, and Postnatal Secondhand Smoking Exposures with the Incidence of Asthma/Atopic Dermatitis in Children: An Epidemiological Study Using Checkup Data of Mothers and Children in Kobe City. Nihon Koshu Eisei Zasshi.

[84] Chao L., Liang W., Zhao X., Liang Z., Wu W., Song J., Ren W. (2024). Maternal Tobacco Exposure during Pregnancy and Atopic Dermatitis in Offspring: A Systematic Review and Meta-Analysis. J. Eur. Acad. Dermatol. Venereol..

[85] Kim S.Y., Sim S., Choi H.G. (2017). Atopic Dermatitis Is Associated with Active and Passive Cigarette Smoking in Adolescents. PLoS ONE.

[86] Lee C.H., Chuang H.Y., Hong C.H., Huang S.K., Chang Y.C., Ko Y.C., Yu H.S. (2011). Lifetime Exposure to Cigarette Smoking and the Development of Adult-Onset Atopic Dermatitis. Br. J. Dermatol..

[87] Moon H.M., Kim Y., Kwak Y., Kim K. (2018). Association between Smoking Type and Prevalence of Atopic Dermatitis and Asthma in Men and Women. Int. J. Nurs. Pract..

[88] Bawany F., Northcott C.A., Beck L.A., Pigeon W.R. (2020). Sleep Disturbances and Atopic Dermatitis: Relationships, Methods for Assessment, and Therapies. J. Allergy Clin. Immunol. Pract..

[89] Samynathan A., Fishbein A.B., Abbott S.M., Booster G.D., Zee P.C., Sheldon S.H., Yosipovitch G., Silverberg J.I. (2024). Assessment and Management of Sleep Disturbances in Atopic Dermatitis: A Review. Dermatitis.

[90] Guo M., Su J., Zheng S., Chen B. (2023). Objective Sleep in Atopic Dermatitis: A Meta-Analysis. Dermatitis.

[91] Lee D.G., Gui X.Y., Mukovozov I., Fleming P., Lynde C. (2023). Sleep Disturbances in Children With Atopic Dermatitis: A Scoping Review. J. Cutan. Med. Surg..

[92] Cameron S., Donnelly A., Broderick C., Arichi T., Bartsch U., Dazzan P., Elbeling J., Godfrey E., Gringras P., Heathcote L.C. (2024). Mind and Skin: Exploring the Links between Inflammation, Sleep Disturbance and Neurocognitive Function in Patients with Atopic Dermatitis. Allergy Eur. J. Allergy Clin. Immunol..

[93] Zhang H., Wang M., Zhao X., Wang Y., Chen X., Su J. (2024). Role of Stress in Skin Diseases: A Neuroendocrine-Immune Interaction View. Brain Behav. Immun..

[94] Zhao Q., Tominaga M., Toyama S., Komiya E., Tobita T., Morita M., Zuo Y., Honda K., Kamata Y., Takamori K. (2024). Effects of Psychological Stress on Spontaneous Itch and Mechanical Alloknesis of Atopic Dermatitis. Acta Derm. Venereol..

[95] Wu Q., Liu S., Li Z., Jin Y., Li X., Han J., Shi C., Shen X., Xia S., Wang J. (2025). FGF-21 Fusion Proteins Ameliorate Atopic Dermatitis by Inhibiting the TLR/TSLP Signaling Pathway: Anti-Inflammatory and Skin Barrier Repair Effects. Int. Immunopharmacol..

[96] Kim J.E., Kim H.S. (2019). Microbiome of the Skin and Gut in Atopic Dermatitis (Ad): Understanding the Pathophysiology and Finding Novel Management Strategies. J. Clin. Med..

[97] Suleman M., Moltrasio C., Tricarico P.M., Marzano A.V., Crovella S. (2024). Natural Compounds Targeting Thymic Stromal Lymphopoietin (TSLP): A Promising Therapeutic Strategy for Atopic Dermatitis. Biomolecules.

[98] Kim J., Kim B.E., Leung D.Y.M. (2019). Pathophysiology of Atopic Dermatitis: Clinical Implications. Allergy Asthma Proc..

[99] Mohammad S., Karim M.R., Iqbal S., Lee J.H., Mathiyalagan R., Kim Y.J., Yang D.U., Yang D.C. (2024). Atopic Dermatitis: Pathophysiology, Microbiota, and Metabolome—A Comprehensive Review. Microbiol. Res..

[100] Nakashima C., Yanagihara S., Otsuka A. (2022). Innovation in the Treatment of Atopic Dermatitis: Emerging Topical and Oral Janus Kinase Inhibitors. Allergol. Int..

[101] Kaczmarska A., Kwiatkowska D., Skrzypek K.K., Kowalewski Z.T., Jaworecka K., Reich A. (2023). Pathomechanism of Pruritus in Psoriasis and Atopic Dermatitis: Novel Approaches, Similarities and Differences. Int. J. Mol. Sci..

[102] Prados-Carmona A., Husein-ElAhmed H., Navarro-Triviño F.J., Ruiz-Villaverde R. (2025). From Pathways to Patients in Atopic Dermatitis: Advanced Systemic Therapies. Int. J. Mol. Sci..

[103] Fan X., Liu Z., Yang W., Zhang H., Zhong H., Pang Y., Ye X., Wu C., Li L. (2025). Advances in Atopic Dermatitis Treatment: From Pathogenesis to Natural Product-Based Therapies. Phyther. Res..

[104] Methé B.A., Nelson K.E., Pop M., Creasy H.H., Giglio M.G., Huttenhower C., Gevers D., Petrosino J.F., Abubucker S., Badger J.H. (2012). A Framework for Human Microbiome Research. Nature.

[105] Moniaga C.S., Tominaga M., Takamori K. (2022). An Altered Skin and Gut Microbiota Are Involved in the Modulation of Itch in Atopic Dermatitis. Cells.

[106] Kalashnikova I.G., Nekrasova A.I., Korobeynikova A.V., Bobrova M.M., Ashniev G.A., Bakoev S.Y., Zagainova A.V., Lukashina M.V., Tolkacheva L.R., Petryaikina E.S. (2024). The Association between Gut Microbiota and Serum Biomarkers in Children with Atopic Dermatitis. Biomedicines.

[107] Depner M., Taft D.H., Peschel S., Roduit C., Karvonen A.M., Barnig C., Divaret-Chauveau A., Riedler J., Pekkanen J., Schmausser-Hechfellner E. (2025). The Janus Face of *Bifidobacterium* in the Development of Atopic Eczema: A Role for Compositional Maturation. Pediatr. Allergy Immunol..

[108] Alam M.J., Xie L., Yap Y.-A., Marques F.Z., Robert R. (2022). Manipulating Microbiota to Treat Atopic Dermatitis: Functions and Therapies. Pathogens.

[109] Huang Z., Lu T., Lin J., Ding Q., Li X., Lin L. (2025). Exploring Causal Relationships Between Gut Microbiota, Inflammatory Cytokines, and Inflammatory Dermatoses: A Mendelian Randomization Study. Clin. Cosmet. Investig. Dermatol..

[110] Hou T., Sun X., Zhu J., Hon K.-L., Jiang P., Chu I.M.-T., Tsang M.S.-M., Lam C.W.-K., Zeng H., Wong C.-K. (2020). IL-37 Ameliorating Allergic Inflammation in Atopic Dermatitis Through Regulating Microbiota and AMPK-MTOR Signaling Pathway-Modulated Autophagy Mechanism. Front. Immunol..

[111] Bylund S., Von Kobyletzki L.B., Svalstedt M., Svensson Å. (2020). Prevalence and Incidence of Atopic Dermatitis: A Systematic Review. Acta Derm. Venereol..

[112] Forno E., Onderdonk A.B., McCracken J., Litonjua A.A., Laskey D., Delaney M.L., DuBois A.M., Gold D.R., Ryan L.M., Weiss S.T. (2008). Diversity of the Gut Microbiota and Eczema in Early Life. Clin. Mol. Allergy.

[113] Wang M., Karlsson C., Olsson C., Adlerberth I., Wold A.E., Strachan D.P., Martricardi P.M., Aberg N., Perkin M.R., Tripodi S. (2008). Reduced Diversity in the Early Fecal Microbiota of Infants with Atopic Eczema. J. Allergy Clin. Immunol..

[114] Abrahamsson T.R., Jakobsson H.E., Andersson A.F., Björkstén B., Engstrand L., Jenmalm M.C. (2012). Low Diversity of the Gut Microbiota in Infants with Atopic Eczema. J. Allergy Clin. Immunol..

[115] Lee E., Lee S.-Y., Kang M.-J., Kim K., Won S., Kim B.-J., Choi K.Y., Kim B.-S., Cho H.-J., Kim Y. (2016). Clostridia in the Gut and Onset of Atopic Dermatitis via Eosinophilic Inflammation. Ann. Allergy. Asthma Immunol..

[116] Xue Y., Zhang L., Chen Y., Wang H., Xie J. (2023). Gut Microbiota and Atopic Dermatitis: A Two-Sample Mendelian Randomization Study. Front. Med..

[117] Penders J., Thijs C., Van Den Brandt P.A., Kummeling I., Snijders B., Stelma F., Adams H., Van Ree R., Stobberingh E.E. (2007). Gut Microbiota Composition and Development of Atopic Manifestations in Infancy: The KOALA Birth Cohort Study. Gut.

[118] Hong P.-Y., Lee B.W., Aw M., Shek L.P.C., Yap G.C., Chua K.Y., Liu W.-T. (2010). Comparative Analysis of Fecal Microbiota in Infants with and without Eczema. PLoS ONE.

[119] Kalliomäki M., Kirjavainen P., Eerola E., Kero P., Salminen S., Isolauri E. (2001). Distinct Patterns of Neonatal Gut Microflora in Infants in Whom Atopy Was and Was Not Developing. J. Allergy Clin. Immunol..

[120] Zheng H., Liang H., Wang Y., Miao M., Shi T., Yang F., Liu E., Yuan W., Ji Z.-S., Li D.-K. (2016). Altered Gut Microbiota Composition Associated with Eczema in Infants. PLoS ONE.

[121] Gore C., Munro K., Lay C., Bibiloni R., Morris J., Woodcock A., Custovic A., Tannock G.W. (2008). Bifidobacterium Pseudocatenulatum Is Associated with Atopic Eczema: A Nested Case-Control Study Investigating the Fecal Microbiota of Infants. J. Allergy Clin. Immunol..

[122] Song H., Yoo Y., Hwang J., Na Y.-C., Kim H.S. (2016). *Faecalibacterium prausnitzii* Subspecies-Level Dysbiosis in the Human Gut Microbiome Underlying Atopic Dermatitis. J. Allergy Clin. Immunol..

[123] Cait A., Cardenas E., Dimitriu P.A., Amenyogbe N., Dai D., Cait J., Sbihi H., Stiemsma L., Subbarao P., Mandhane P.J. (2019). Reduced Genetic Potential for Butyrate Fermentation in the Gut Microbiome of Infants Who Develop Allergic Sensitization. J. Allergy Clin. Immunol..

[124] Seo S.C., Ahn S.H., Ri S., Yoon Y., Byeon J.H., Kim S.H., Yoon W., Yoo Y. (2018). Elevated Fecal Calprotectin Levels Are Associated with Severity of Atopic Dermatitis in Children. Asian Pac. J. Allergy Immunol..

[125] Keeney K.M., Yurist-Doutsch S., Arrieta M.C., Finlay B.B. (2014). Effects of Antibiotics on Human Microbiota and Subsequent Disease. Annu. Rev. Microbiol..

[126] Koh L.F., Ong R.Y., Common J.E. (2022). Skin Microbiome of Atopic Dermatitis. Allergol. Int..

[127] Lee M.J., Park Y.M., Kim B., Tae I.H., Kim N.E., Pranata M., Kim T., Won S., Kang N.J., Lee Y.K. (2022). Disordered Development of Gut Microbiome Interferes with the Establishment of the Gut Ecosystem during Early Childhood with Atopic Dermatitis. Gut Microbes.

[128] Bratosin F., Facchin S., Bertin L., Bonazzi E., Lorenzon G., De Barba C., Barberio B., Zingone F., Maniero D., Scarpa M. (2024). Short-Chain Fatty Acids and Human Health: From Metabolic Pathways to Current Therapeutic Implications. Life.

[129] Guo W., Liu J., Sun J., Gong Q., Ma H., Kan X., Cao Y., Wang J., Fu S. (2019). Butyrate Alleviates Oxidative Stress by Regulating NRF_2_ Nuclear Accumulation and H_3_K_9_/_14_ Acetylation via GPR_109A_ in Bovine Mammary Epithelial Cells and Mammary Glands. Free Radic. Biol. Med..

[130] Silveira A.K., Gomes H.M., Fröhlich N.T., Possa L., Santos L., Kessler F., Martins A., Rodrigues M.S., De Oliveira J., do Nascimento N.D. (2023). Sodium Butyrate Protects Against Intestinal Oxidative Damage and Neuroinflammation in the Prefrontal Cortex of Ulcerative Colitis Mice Model. Immunol. Investig..

[131] Duc Huy Ta L., Chun Yip Chan J., Chin Yap G., Purbojati R.W., Drautz-Moses D.I., Michelle Koh Y., Jing Xuan Tay C., Huang C.-H., Yan Qin Kioh D., Yun Woon J. (2020). A Compromised Developmental Trajectory of the Infant Gut Microbiome and Metabolome in Atopic Eczema. Gut Microbes.

[132] Kang M., Jung J.H., Kim J.Y., Hong S.H., Her Y. (2023). Therapeutic and Preventive Effect of Orally Administered Prebiotics on Atopic Dermatitis in a Mouse Model. Allergy. Asthma Immunol. Res..

[133] Yu J., Luo Y., Zhu Z., Zhou Y., Sun L., Gao J., Sun J., Wang G., Yao X., Li W. (2019). A Tryptophan Metabolite of the Skin Microbiota Attenuates Inflammation in Patients with Atopic Dermatitis through the Aryl Hydrocarbon Receptor. J. Allergy Clin. Immunol..

[134] Imlay J.A. (2008). Cellular Defenses against Superoxide and Hydrogen Peroxide. Annu. Rev. Biochem..

[135] Bertino L., Guarneri F., Cannavò S.P., Casciaro M., Pioggia G., Gangemi S. (2020). Antioxidants Oxidative Stress and Atopic Dermatitis. Antioxidants.

[136] Belkaid Y., Hand T.W. (2014). Role of the Microbiota in Immunity and Inflammation. Cell.

[137] Díez-Madueño K., De La P., Dobao C., Torres-Rojas I., Fernández-Gosende M., Hidalgo-Cantabrana C., Coto-Segura P. (2024). Gut Dysbiosis and Adult Atopic Dermatitis: A Systematic Review. J. Clin. Med..

[138] Dokoshi T., Chen Y., Cavagnero K.J., Rahman G., Hakim D., Brinton S., Schwarz H., Brown E.A., O’Neill A., Nakamura Y. (2024). Dermal Injury Drives a Skin to Gut Axis That Disrupts the Intestinal Microbiome and Intestinal Immune Homeostasis in Mice. Nat. Commun..

[139] Jimenez-Sanchez M., Celiberto L.S., Yang H., Sham H.P., Vallance B.A. (2025). The Gut-Skin Axis: A Bi-Directional, Microbiota-Driven Relationship with Therapeutic Potential. Gut Microbes.

[140] Kurhaluk N., Kamiński P., Tkaczenko H. (2025). Role of Gut Microbiota in Modulating Oxidative Stress Induced by Environmental Factors. Cell. Physiol. Biochem..

[141] Roy S., Dhaneshwar S. (2023). Role of Prebiotics, Probiotics, and Synbiotics in Management of Inflammatory Bowel Disease: Current Perspectives. World J. Gastroenterol..

[142] Hill C., Guarner F., Reid G., Gibson G.R., Merenstein D.J., Pot B., Morelli L., Canani R.B., Flint H.J., Salminen S. (2014). The International Scientific Association for Probiotics and Prebiotics Consensus Statement on the Scope and Appropriate Use of the Term Probiotic. Nat. Rev. Gastroenterol. Hepatol..

[143] Kavyani B., Ahmadi S., Nabizadeh E., Abdi M. (2024). Anti-Oxidative Activity of Probiotics; Focused on Cardiovascular Disease, Cancer, Aging, and Obesity. Microb. Pathog..

[144] Sánchez B., Delgado S., Blanco-Míguez A., Lourenço A., Gueimonde M., Margolles A. (2017). Probiotics, Gut Microbiota, and Their Influence on Host Health and Disease. Mol. Nutr. Food Res..

[145] Latif A., Shehzad A., Niazi S., Zahid A., Ashraf W., Iqbal M.W., Rehman A., Riaz T., Aadil R.M., Khan I.M. (2023). Probiotics: Mechanism of Action, Health Benefits and Their Application in Food Industries. Front. Microbiol..

[146] Rusu E., Enache G., Cursaru R., Alexescu A., Radu R., Onila O., Cavallioti T., Rusu F., Posea M., Jinga M. (2019). Prebiotics and Probiotics in Atopic Dermatitis. Exp. Ther. Med..

[147] Wang L., Xu L. (2025). The Impact of Prebiotics, Probiotics and Synbiotics on the Prevention and Treatment of Atopic Dermatitis in Children: An Umbrella Meta-Analysis. Front. Pediatr..

[148] Feng T., Wang J. (2020). Oxidative Stress Tolerance and Antioxidant Capacity of Lactic Acid Bacteria as Probiotic: A Systematic Review. Gut Microbes.

[149] Hoffmann A., Kleniewska P., Pawliczak R. (2019). Antioxidative Activity of Probiotics. Arch. Med. Sci..

[150] Ghavami A., Khorvash F., Khalesi S., Heidari Z., Askari G. (2021). The Effects of Synbiotic Supplementation on Oxidative Stress and Clinical Symptoms in Women with Migraine: A Double-blind, Placebo-controlled, Randomized Trial. J. Funct. Foods.

[151] Musazadeh V., Faghfouri A.H., Zarezadeh M., Pakmehr A., Moghaddam P.T., Hamedi-Kalajahi F., Jahandideh A., Ghoreishi Z. (2023). Remarkable Impacts of Probiotics Supplementation in Enhancing of the Antioxidant Status: Results of an Umbrella Meta-Analysis. Front. Nutr..

[152] Litus O., Derkach N., Litus V., Bisyuk Y., Lytvynenko B. (2019). Efficacy of Probiotic Therapy on Atopic Dermatitis in Adults Depends on the C-159T Polymorphism of the CD14 Receptor Gene—A Pilot Study. Open Access Maced. J. Med. Sci..

[153] Wang F., Wu F., Chen H., Tang B. (2023). The Effect of Probiotics in the Prevention of Atopic Dermatitis in Children: A Systematic Review and Meta-Analysis. Transl. Pediatr..

[154] Zhong L., Su J., Zhou X., Wan H. (2025). Probiotics Supplements for the Prevention of Atopic Dermatitis in Children: An Umbrella Review. Front. Nutr..

[155] Kleniewska P., Pawliczak R. (2017). Influence of Synbiotics on Selected Oxidative Stress Parameters. Oxid. Med. Cell. Longev..

[156] Fanfaret I.S., Boda D., Ion L.M., Hosseyni D., Leru P., Ali S., Corcea S., Bumbacea R. (2021). Probiotics and Prebiotics in Atopic Dermatitis: Pros and Cons (Review). Exp. Ther. Med..

[157] Gérard P. (2015). Gut Microbiota and Obesity. Cell. Mol. Life Sci..

[158] Yoo S., Jung S.C., Kwak K., Kim J.S. (2024). The Role of Prebiotics in Modulating Gut Microbiota: Implications for Human Health. Int. J. Mol. Sci..

[159] Chang Y.S., Trivedi M.K., Jha A., Lin Y.F., Dimaano L., García-Romero M.T. (2016). Synbiotics for Prevention and Treatment of Atopic Dermatitis: A Meta-Analysis of Randomized Clinical Trials. JAMA Pediatr..

[160] Ozma M.A., Abbasi A., Akrami S., Lahouty M., Shahbazi N., Ganbarov K., Pagliano P., Sabahi S., Köse Ş., Yousefi M. (2022). Postbiotics as the Key Mediators of the Gut Microbiota-Host Interactions. Le Infez. Med..

[161] Thorakkattu P., Khanashyam A.C., Shah K., Babu K.S., Mundanat A.S., Deliephan A., Deokar G.S., Santivarangkna C., Nirmal N.P. (2022). Postbiotics: Current Trends in Food and Pharmaceutical Industry. Foods.

[162] Rezaie N., Aghamohammad S., Khiavi E.H.A.G., Talebi M., Pourshafie M.R., Rohani M. (2025). The Analysis and Comparison of Anti-Inflammatory and Antioxidant Characteristics of Postbiotic and Paraprobiotic Derived From Novel Native Probiotic Cocktail in DSS-Induced Colitic Mice. Food Sci. Nutr..

[163] Ji J., Jin W., Liu S.J., Jiao Z., Li X. (2023). Probiotics, Prebiotics, and Postbiotics in Health and Disease. MedComm.

[164] Lee Y.-S., Noh D.-I., Lee S.-J., Jeon M.-H., Kim Y.-R., Jang W.J., Lee E.-W. (2025). Anti-Inflammatory and Barrier-Restoring Effects of Heat-Killed Lactiplantibacillus Plantarum Postbiotics in an in Vitro Model of Atopic Dermatitis. J. Appl. Microbiol..

[165] Tanojo N., Citrashanty I., Utomo B., Listiawan Y., Ervianti E. (2023). Oral Postbiotics Derived from *Lactobacillus* sp. in Treatment of Atopic Dermatitis: A Meta-Analysis. Acta Dermatovenerol..

[166] Cheng Y.W., Fischer M. (2023). Fecal Microbiota Transplantation. Clin. Colon. Rectal Surg..

[167] Liu M., Ma J., Xu J., Huangfu W., Zhang Y., Ali Q., Liu B., Li D., Cui Y., Wang Z. (2024). Fecal Microbiota Transplantation Alleviates Intestinal Inflammatory Diarrhea Caused by Oxidative Stress and Pyroptosis via Reducing Gut Microbiota-Derived Lipopolysaccharides. Int. J. Biol. Macromol..

[168] Liu X., Liu C., Qian X., Zhang S., Yao Z., Chai Y., Shi Q., Yang W., Wang Q., Zhang L. (2025). Fecal Microbiota Transplantation Alleviated Heat-Induced Colonic Tissue Damage, Epithelial Apoptosis, and Oxidative Stress. Appl. Environ. Microbiol..

[169] Kim J.H., Kim K., Kim W. (2021). Gut Microbiota Restoration through Fecal Microbiota Transplantation: A New Atopic Dermatitis Therapy. Exp. Mol. Med..

[170] Wu M., Chen X., Lu Q., Yao X. (2024). Fecal Microbiota Transplantation for the Treatment of Chronic Inflammatory Skin Diseases. Heliyon.

[171] Ouwehand A.C. (2017). A Review of Dose-Responses of Probiotics in Human Studies. Benef. Microbes.

[172] Bevilacqua A., Campaniello D., Speranza B., Racioppo A., Sinigaglia M., Corbo M.R. (2024). An Update on Prebiotics and on Their Health Effects. Foods.

[173] Ma L., Tu H., Chen T. (2023). Postbiotics in Human Health: A Narrative Review. Nutrients.

[174] Sergeev I.N., Aljutaily T., Walton G., Huarte E. (2020). Effects of Synbiotic Supplement on Human Gut Microbiota, Body Composition and Weight Loss in Obesity. Nutrients.

[175] Rapoport E.A., Baig M., Puli S.R. (2022). Adverse Events in Fecal Microbiota Transplantation: A Systematic Review and Meta-Analysis. Ann. Gastroenterol..

[176] Jeskey J., Kurien C., Blunk H., Sehmi K., Areti S., Nguyen D., Hostoffer R. (2024). Atopic Dermatitis: A Review of Diagnosis and Treatment. J. Pediatr. Pharmacol. Ther..

[177] Silverberg J.I., Wollenberg A., Reich A., Thaçi D., Legat F.J., Papp K.A., Stein Gold L., Bouaziz J.D., Pink A.E., Carrascosa J.M. (2024). Nemolizumab with Concomitant Topical Therapy in Adolescents and Adults with Moderate-to-Severe Atopic Dermatitis (ARCADIA 1 and ARCADIA 2): Results from Two Replicate, Double-Blind, Randomised Controlled Phase 3 Trials. Lancet.

[178] Lee H.W., Ju Y.J., Choi S., Rhew K., Sevilleno S.S., Choi M.S. (2025). Atopic Dermatitis Management: From Conventional Therapies to Biomarker-Driven Treatment Approaches. Biomol. Ther..

[179] Labib A., Does A.V., Yosipovitch G. (2022). Nemolizumab for Atopic Dermatitis. Drugs Today.

[180] Yilmaz O., Torres T. (2024). Extended Half-Life Antibodies: A Narrative Review of a New Approach in the Management of Atopic Dermatitis. Dermatol. Ther..

[181] Ryguła I., Pikiewicz W., Kaminiów K. (2023). Novel Janus Kinase Inhibitors in the Treatment of Dermatologic Conditions. Molecules.

[182] Papp K., Szepietowski J.C., Kircik L., Toth D., Eichenfield L.F., Leung D.Y.M., Forman S.B., Venturanza M.E., Sun K., Kuligowski M.E. (2021). Efficacy and Safety of Ruxolitinib Cream for the Treatment of Atopic Dermatitis: Results from 2 Phase 3, Randomized, Double-Blind Studies. J. Am. Acad. Dermatol..

[183] Bieber T., Simpson E.L., Silverberg J.I., Thaçi D., Paul C., Pink A.E., Kataoka Y., Chu C.-Y., DiBonaventura M., Rojo R. (2021). Abrocitinib versus Placebo or Dupilumab for Atopic Dermatitis. N. Engl. J. Med..

[184] Silverberg J.I., Simpson E.L., Wollenberg A., Bissonnette R., Kabashima K., Delozier A.M., Sun L., Cardillo T., Nunes F.P., Reich K. (2021). Long-Term Efficacy of Baricitinib in Adults With Moderate to Severe Atopic Dermatitis Who Were Treatment Responders or Partial Responders: An Extension Study of 2 Randomized Clinical Trials. JAMA Dermatol..

[185] Whayne T.F., Saha S.P., Mukherjee D. (2016). Antioxidants in the Practice of Medicine; What Should the Clinician Know?. Cardiovasc. Hematol. Disord. Drug Targets.

[186] Poljsak B., Šuput D., Milisav I. (2013). Achieving the Balance between ROS and Antioxidants: When to Use the Synthetic Antioxidants. Oxid. Med. Cell. Longev..

[187] De Simoni E., Candelora M., Belleggia S., Rizzetto G., Molinelli E., Capodaglio I., Ferretti G., Bacchetti T., Offidani A., Simonetti O. (2024). Role of Antioxidants Supplementation in the Treatment of Atopic Dermatitis: A Critical Narrative Review. Front. Nutr..

[188] Yang H., Chen J.S., Luo X.Y., Wang H. (2022). Efficacy and Safety Profile of Antioxidants in the Treatment of Atopic Dermatitis: A Systematic Review and Meta-Analysis of Randomized Controlled Trials. Dermatol. Ther..

[189] Chauhan K., Shahrokhi M., Huecker M.R. (2025). Vitamin D. StatPearls [Internet].

[190] Blady K., Pomianowski B., Strugała M., Smółka L., Kursa K., Stanek A. (2025). Vitamin D in Atopic Dermatitis: Role in Disease and Skin Microbiome. Nutrients.

[191] Di Filippo P., Scaparrotta A., Rapino D., Cingolani A., Attanasi M., Petrosino M.I., Chuang K., Di Pillo S., Chiarelli F. (2015). Vitamin D Supplementation Modulates the Immune System and Improves Atopic Dermatitis in Children. Int. Arch. Allergy Immunol..

[192] van Harten R.M., van Woudenbergh E., van Dijk A., Haagsman H.P. (2018). Cathelicidins: Immunomodulatory Antimicrobials. Vaccines.

[193] Nielsen A.Y., Høj S., Thomsen S.F., Meteran H. (2024). Vitamin D Supplementation for Treating Atopic Dermatitis in Children and Adults: A Systematic Review and Meta-Analysis. Nutrients.

[194] Amon U., Baier L., Yaguboglu R., Ennis M., Holick M.F., Amon J. (2018). Serum 25-Hydroxyvitamin D Levels in Patients with Skin Diseases Including Psoriasis, Infections, and Atopic Dermatitis. Dermatoendocrinology.

[195] Jaffary F., Faghihi G., Mokhtarian A., Hosseini S.M. (2015). Effects of Oral Vitamin E on Treatment of Atopic Dermatitis: A Randomized Controlled Trial. J. Res. Med. Sci..

[196] Lee S., Ahn K., Paik H.Y., Chung S.J. (2012). Serum Immunoglobulin E (IgE) Levels and Dietary Intake of Korean Infants and Young Children with Atopic Dermatitis. Nutr. Res. Pract..

[197] Abdullah M., Jamil R.T., Attia F.N. (2023). Vitamin C (Ascorbic Acid). Encycl. Toxicol. Fourth Ed..

[198] Wang K., Jiang H., Li W., Qiang M., Dong T., Li H. (2018). Role of Vitamin C in Skin Diseases. Front. Physiol..

[199] Nassar B., Belhadj-Tahar H., Jin W., Yang G. (2025). A Prospective Open-Label Study of Tolerance and Effectiveness of Sequential Dermocosmetic Treatments Combining Poly-l-Lysine Biovectors With Vitamins A and C. Health Sci. Rep..

[200] Hoppu U., Rinne M., Salo-Väänänen P., Lampi A.M., Piironen V., Isolauri E. (2004). Vitamin C in Breast Milk May Reduce the Risk of Atopy in the Infant. Eur. J. Clin. Nutr..

[201] González-Peña M.A., Ortega-Regules A.E., Anaya de Parrodi C., Lozada-Ramírez J.D. (2023). Chemistry, Occurrence, Properties, Applications, and Encapsulation of Carotenoids—A Review. Plants.

[202] You J.S., Jeon S., Byun Y.J., Koo S., Choi S.S. (2015). Enhanced Biological Activity of Carotenoids Stabilized by Phenyl Groups. Food Chem..

[203] Stanescu C., Chiscop I., Mihalache D., Popa F., Tamas C., Stoleriu G. (2025). Skin Aging and Carotenoids: A Systematic Review of Their Multifaceted Protective Mechanisms. Nutrients.

[204] Miyake Y., Sasaki S., Tanaka K., Hirota Y. (2010). Consumption of Vegetables, Fruit, and Antioxidants during Pregnancy and Wheeze and Eczema in Infants. Allergy.

[205] Inoue Y., Yamamoto Y., Suzuki S., Ochiai S., Eguchi A., Nakano T., Yamaide F., Hasegawa S., Kojima H., Mori C. (2023). Maternal and Infant Serum Carotenoids Are Associated with Infantile Atopic Dermatitis Development. Allergy.

